# Hypoxia‐sensitive LINC01436 is regulated by E2F6 and acts as an oncogene by targeting miR‐30a‐3p in non‐small cell lung cancer

**DOI:** 10.1002/1878-0261.12437

**Published:** 2019-01-30

**Authors:** Shuai Yuan, Ying Xiang, Guilu Wang, Meiyu Zhou, Gang Meng, Qingyun Liu, Zeyao Hu, Chengying Li, Weijia Xie, Na Wu, Long Wu, Tongjian Cai, Xiangyu Ma, Yao Zhang, Zubin Yu, Li Bai, Yafei Li

**Affiliations:** ^1^ Department of Epidemiology College of Preventive Medicine Army Medical University (Third Military Medical University) Chongqing China; ^2^ Department of Epidemiology School of Public Health Guizhou Medical University China; ^3^ Department of Pathology Southwest Hospital Army Medical University (Third Military Medical University) Chongqing China; ^4^ Department of Thoracic Surgery Xinqiao Hospital Army Medical University (Third Military Medical University) Chongqing China; ^5^ Department of Respiratory Disease Xinqiao Hospital Army Medical University (Third Military Medical University) Chongqing China

**Keywords:** EPAS1, hypoxia, LINC01436, long noncoding RNA, microRNA‐30a‐3p, non‐small cell lung cancer

## Abstract

Dysregulation of long noncoding RNA (lncRNA) is known to be involved in numerous human diseases, including lung cancer. However, the precise biological functions of most lncRNA remain to be elucidated. Here, we identified a novel up‐regulated lncRNA, LINC01436 (RefSeq: NR_110419.1), in non‐small cell lung cancer (NSCLC). High expression of LINC01436 was significantly associated with poor overall survival. Notably, LINC01436 expression was transcriptionally repressed by E2F6 under normoxia, and the inhibitory effect was relieved in a hypoxic microenvironment. Gain‐ and loss‐of‐function studies revealed that LINC01436 acted as a proto‐oncogene by promoting lung cancer cell growth, migration and invasion *in vitro*. Xenograft tumor assays in nude mice confirmed that LINC01436 promoted tumor growth and metastasis *in vivo*. Mechanistically, LINC01436 exerted biological functions by acting as a microRNA (miR)‐30a‐3p sponge to regulate the expression of its target gene *EPAS1*. Our findings characterize LINC01436 as a new hypoxia‐sensitive lncRNA with oncogenic function in NSCLC, suggesting that LINC01436 may be a potential biomarker for prognosis and a potential target for treatment.

**Abbreviations**
CCK‐8: cell counting kit‐8
ceRNA: competitive endogenous RNA
CPAT: coding potential assessment tool
CPC: coding potential calculator
EPAS1: endothelial PAS domain‐containing protein 1
FISH: fluorescence *in situ* hybridization
GLUT1: glucose transporter 1
H&E: hematoxylin and eosin
HIF: hypoxia‐inducible factor
lncHIFCAR: long noncoding HIF‐1α co‐activating
lncRNA: long noncoding RNA
mRNA: messenger RNA
miRNA: microRNA
MUT: mutant type
NSCLC: non‐small cell lung cancer
ORF: open reading frame
qRT‐PCR: quantitative real‐time PCR
RACE: rapid amplification of cDNA ends
siRNA: small interfering RNA
TCGA: The Cancer Genome Atlas
TSS: transcription start site
VEGFA: vascular endothelial growth factor A
WB: western blot
WT: wild type

## 1 Introduction

Lung cancer is one of the leading causes of cancer‐related death in China and around the world (Chen *et al*., 2016; Siegel *et al*., 2017). Non‐small cell lung cancer (NSCLC) accounts for about 85% of lung cancer, and adenocarcinoma is the major histological type of NSCLC (Ettinger *et al*., 2013). Despite improvement in early diagnosis and therapeutic response, the overall survival (OS) rate is still < 20% in NSCLC patients (Chen *et al*., 2014). Thus, identification of new effective diagnostic markers and therapeutic targets may provide alternative strategies to reduce lung cancer mortality.

Long noncoding RNA (lncRNA) have been shown to contribute to cancerous phenotypes such as proliferation, growth suppression, motility, immortality, angiogenesis and viability (Fu *et al*., 2017; Lin *et al*., 2017; Schmitt and Chang, 2016). Evidence has indicated that lncRNA function primarily through their interactions with cellular macromolecules such as protein, DNA or RNA (Fang *et al*., 2016; Schmitt and Chang, 2016; Sun *et al*., 2016b; Zhang *et al*., 2018). The competitive endogenous RNA (ceRNA) hypothesis (Salmena *et al*., 2011; Thomson and Dinger, 2016) proposes that lncRNA with shared microRNA (miRNA) binding sites can act as endogenous miRNA sponges and post‐transcriptionally regulate the biological activity of miRNA target genes (Sun *et al*., 2016a; Yang *et al*., 2016).

E2F is a group of genes that codifies a family of transcription factors. E2F family members play a major role in the cell cycle regulation and synthesis of DNA in mammalian cells (Benevolenskaya and Frolov, 2015). A member of the E2F family, E2F6, contains a modular suppression domain and thus was classified as a transcriptional repressor (Cartwright *et al*., 1998). A group of protein‐coding genes related to cellular proliferation, differentiation and cell fate were targeted and repressed by E2F6 (Leseva *et al*., 2013; Velasco *et al*., 2010). However, apart from H19 (Berteaux *et al*., 2005), very few lncRNA have been reported to be regulated by E2F6.

Hypoxia is a hallmark of the tumor microenvironment. It is related to proliferation, metastasis, recurrence and resistance to therapy in many solid tumors (Rey *et al*., 2017). Hypoxia‐inducible factors‐1α and ‐2α (HIF‐1α and HIF‐2α) are two extensively studied HIF that primarily mediate the cellular response to hypoxia (Schito and Semenza, 2016). Several hypoxia‐responsive lncRNA have been reported to play important roles in HIF pathway regulation and tumorigenesis processes (Chang *et al*., 2016). One recent study revealed that long noncoding HIF‐1α co‐activating RNA (lncHIFCAR) could modulate hypoxia signaling pathways by acting as an HIF‐1α co‐activator (Shih *et al*., 2017). However, how lncRNA interact with hypoxic regulators in tumor progression remains largely unclear. Thus, further investigations should be carried out on the role of hypoxia‐related lncRNA as well as their functional mechanisms in lung cancer.

In the present study, we perform a microarray analysis to screen lncRNA that are differentially expressed in NSCLC. The quantitative real‐time PCR (qRT‐PCR) analysis confirms LINC01436 is one of the top up‐regulated lncRNA. LINC01436 is a long intergenic noncoding RNA located at chromosome 21q22.12. We subsequently focus on the functional role and potential mechanism of LINC01436. Its high expression is associated with poor prognosis in NSCLC. We further demonstrate here that abnormally high expression of LINC01436 promotes growth and metastasis of NSCLC. LINC01436 functions as a ceRNA via competitively binding miR‐30a‐3p and regulating target gene endothelial PAS domain‐containing protein 1 (EPAS1). The expression of LINC01436 is inhibited by transcription factor E2F6. Moreover, hypoxia can relieve the inhibitory effect of E2F6 on LINC01436 and rescue the oncogenic activity of LINC01436.

## 2 Materials and methods

### 2.1 Cell culture and treatment

The lung cancer cell lines A549, SPCA1 and H1299 were obtained from the Cell Bank of the Chinese Academy of Science (Shanghai, China) and the American Type Culture Collection (Manassas, VA, USA), cultured in RPMI‐1640 (HyClone, Logan, UT, USA) or F12K (Gibco, Life Technology, Carlsbad, CA, USA) supplemented with 10% fetal bovine serum (HyClone). For hypoxia treatment, cells were cultured in the presence of 1% O_2_ for 24 and 48 h or treated with different doses of CoCl_2_ (100–500 μm; Sigma, St. Louis, MO, USA) for 48 h.

### 2.2 Human tissue samples

Lung cancer patients were enrolled at Xinqiao Hospital of the Third Military Medical University in Chongqing, China. Tissue specimens were collected prior to any radiation or chemotherapy during operation. The fresh‐frozen lung tumors and matched normal lung tissues were sectioned and reviewed by a pathologist to confirm the diagnosis of lung cancer, histological grade, tumor purity, and lack of tumor contamination in the normal lung. Tumor samples with ≥ 70% tumor‐cell content and matched normal lung tissues were used in the study. Five cases were used for lncRNA and miRNA microarray analysis, and 100 cases were analyzed by qRT‐PCR (Tables S1 and S2). All patients provided informed written consent. The research protocol was approved by the ethics committee of the Third Military Medical University. The study methodologies conformed to the standards set by the Declaration of Helsinki.

### 2.3 Expression microarray analysis of lncRNA and miRNA

Total RNA was extracted with RNeasy kit (Qiagen, Madison, WI, USA) according to the manufacturer's protocol. Expression of genomic lncRNA was detected using the Affymetrix GeneChip Human Transcriptome Array (HTA2.0; Affymetrix, Santa Clara, CA, USA). Expression of genomic miRNA was detected using the GeneChip® microRNA4.0 array (Affymetrix). The microarray experiments were performed by the Gminix Corporation (Shanghai, China).

### 2.4 qRT‐PCR analysis

Total RNA was extracted using TRIzol reagent (TaKaRa, Dalian, China) and cDNA were synthesized using PrimeScript™ RT reagent Kit with gDNA Eraser (TaKaRa). Nuclear and cytoplasmic RNA were isolated using PARIS™ Kit (Invitrogen, Carlsbad, CA, USA). Real‐time PCR was performed using the SYBR Premix Ex Taq (TaKaRa) following the manufacturer's instructions. Results were normalized to the housekeeping gene β*‐actin*. Primers sequences were provided in Table S3.

### 2.5 5′‐Rapid amplification of cDNA ends

5′‐Rapid amplification of cDNA ends (RACE) was performed following the protocol of the 5′‐RACE System for RACE (Invitrogen). Briefly, first‐strand cDNA was synthesized using a gene‐specific primer. The original messenger RNA (mRNA) template was removed by RNase H and a homopolymeric tail was then added to the 3′‐end of the cDNA using TdT and dCTP. A nested PCR was then carried out to amplify cDNA from the 5′‐end. Primers sequences are provided in Table S3.

### 2.6 Plasmid construction and cell transfection

To construct plasmids expressing LINC01436 and E2F6, the full‐length human LINC01436 sequence (RefSeq: NR_110419.1) and E2F6 (RefSeq: NM_198256.3) were synthesized and subcloned into the pcDNA3.1 vector (Invitrogen). The plasmids were transfected using Lipofectamine 2000 Reagent (Invitrogen). The stably transfected cells were screened under G418 (Sangon Biotech, Shanghai, China). For gene knockdown, 100 pmol small interfering RNA (siRNA; GenePharma, Shanghai, China) were transfected into cells with Lipofectamine 2000 (Invitrogen) in six‐well plates. LINC01436 and E2F6 siRNA sequences are shown in Table S3. MiR‐30a‐3p mimics and inhibitors were purchased from GenePharma (Table S3). To avoid the interference of miR‐30a‐5p (the reverse complementary sequence of miR‐30a‐3p) (Liu *et al*., 2017), we used single strand miR‐30a‐3p mimics for study.

### 2.7 Cell counting kit‐8 assay, colony formation assay, cell migration and invasion assays *in vitro*

Cell counting kit‐8 (CCK‐8) assay, colony formation assay, cell migration and invasion assays were described previously (Yuan *et al*., 2016).

### 2.8 Animal experiments *in vivo*

For *in vivo* tumorigenicity, eight male BALB/c‐nude mice (4 weeks old) were randomly divided into two groups, with four mice in each group. Stably transfected A549 cells (5.0 × 10^6^) were injected subcutaneously into the left flanks of the nude mice. The tumor volume was calculated using the equation *V* = 0.5 × *D* × *d* ^2^, where *V =* volume; *D* = longitudinal diameter and *d* = latitudinal diameter. We observed the tumor growth for 5 weeks.

For the metastasis model, 10 male BALB/c‐nude mice (4 weeks old) were randomly divided into two groups, with five mice in each group. Stably transfected A549 cells (1 × 10^6^) were injected into the tail veins of the nude mice. The mice were sacrificed 5 weeks after injection and the lungs were removed for further analysis. All experimental animal procedures were approved by the Institutional Animal Care and Use Committee of Third Military Medical University.

### 2.9 Luciferase reporter assay

To evaluate the miRNA‐lncRNA/mRNA interaction by luciferase reporter assay, the miR‐30a‐3p binding sites of LINC01436 or EPAS1 were inserted into the pMIR‐RB‐REPORT™ vector (Ribobio, Guangzhou, China). The reconstituted plasmid was named LINC01436‐wild type (WT) or EPAS1‐WT. The miR‐30a‐3p target site mutations were introduced and inserted into the pMIR‐RB‐REPORT™ vector (Ribobio), which was named LINC01436‐mutant type (MUT) or EPAS1‐MUT. The plasmid construction was performed by Ribobio Corporation. A549 cells were seeded into 24‐well plates (2 × 10^4^ cells per well) in triplicate for each group. After overnight incubation, cells were co‐transfected with reconstituted plasmid and miR‐30a‐3p mimics. Firefly and Renilla luciferase activities were measured 48 h after transfection using the Dual‐Luciferase Assay System (Promega, Madison, WI, USA). The relative luciferase activity was calculated using Renilla/Firefly luciferase activity.

To evaluate the binding of E2F6 to the promoter of LINC01436, the LINC01436 promoter sequence (−354 to +133 bp) was synthesized and subcloned into the luciferase reporter vector pGL3‐basic (Promega). The reconstituted plasmid was named pGL3‐basic‐LINC01436. The plasmid construction was performed by Sangon Corporation. H1299 cells were seeded into 24‐well plates (4 × 10^4^ cells per well) and co‐transfected with reconstituted plasmid and pcDNA3.1‐E2F6 (or si‐E2F6) as well as pRL‐SV40 Renilla luciferase plasmid (Promega) for internal control. The luciferase activities were measured 48 h after transfection and the relative luciferase activity was calculated using Firefly/Renilla luciferase activity.

### 2.10 Western blot and immunohistochemistry

Western blot (WB) was performed as described previously (Yuan *et al*., 2016). The antibodies used in this study were rabbit monoclonal antibody to EPAS1 (HIF‐2‐alpha; 1 : 1000; Abcam Inc., Cambridge, MA, USA), rabbit monoclonal antibody to E2F6 (1 : 1000; Abcam) and rabbit polyclonal antibody to β*‐actin* (1 : 5000; Abcam). Immunohistochemistry was performed as described in Liu *et al*. (2010) using the rabbit monoclonal antibody to Ki‐67 (1 : 500; Abcam).

### 2.11 CHIP assay

CHIP assay was performed following the protocol of ChIP Assay Kit (Beyotime Institute of Biotechnology, Jiangsu, China). Briefly, cross‐linked chromatin was prepared with 1% formaldehyde for 10 min at 37 °C and the DNA was shredded to an average length of 200–1000 bp by sonication. Immunoprecipitation was conducted with rabbit polyclonal antibody to E2F6 (Abcam) or IgG control. Precipitated DNA was amplified by PCR. Primer sequences are provided in Table S3.

### 2.12 RNA fluorescence *in situ* hybridization

Cy3‐labeled LINC01436 probes were synthesized by RiboBio. RNA fluorescence *in situ* hybridization (FISH) was performed as described by Zhou *et al*. (2015) using RNA FISH kit (RiboBio) following the manufacturer's instructions. U6 snRNA and 18S rRNA probes were purchased from RiboBio and were used as nuclear and cytoplasmic localization controls, respectively.

### 2.13 Bioinformatics analysis

LINC01436 expression data in The Cancer Genome Atlas (TCGA) lung cancer dataset were downloaded from the TANRIC website (http://ibl.mdanderson.org/tanric/_design/basic/index.html). Clinical data from TCGA (http://cancergenome.nih.gov/) were used to assess the clinical significance of LINC01436 in NSCLC. LINC01436 expression levels were classified as high or low expression using median gene expression value as the cutoff. The survival curves were calculated using the Kaplan–Meier method and the differences were evaluated using the log‐rank test. Univariate and multivariate Cox proportional hazards regression models were used to identify the independent factors of OS for NSCLC patients. The miRNA mature strand expression data of TCGA lung cancer (*n* = 875) from UCSC Xena (https://xenabrowser.net/heatmap/) were used to compare differential miRNA expression in tumor (*n* = 786) and normal tissues (*n* = 89).

### 2.14 Statistical analysis

Statistical analyses were performed using spss 19.0 software (SPSS, Inc., Chicago, IL, USA). Cell growth and migration results were evaluated using the two‐tailed Student's *t*‐test. Gene expression analyses were evaluated using the two‐tailed Student's *t*‐test or Mann–Whitney *U*‐test. Pearson correlation was performed to analyze the correlation between miRNA and mRNA expressions. A two‐sided *P*‐value < 0.05 was taken as statistically significant.

## 3 Results

### 3.1 LINC01436 expression is significantly up‐regulated and associated with overall survival in NSCLC

**Figure 1:**
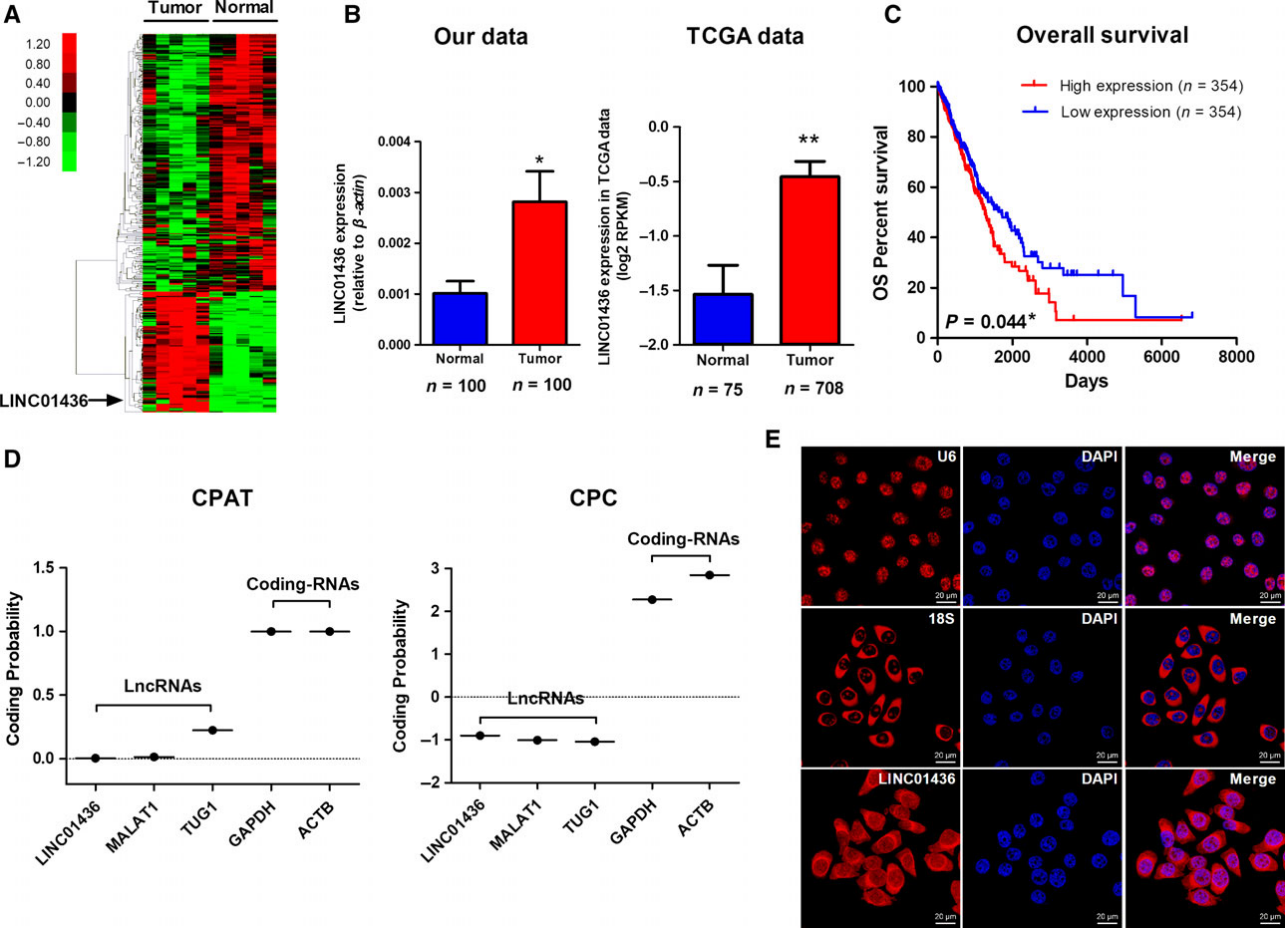
LINC01436 expression is up‐regulated in NSCLC and its characterization. (A) Genome‐wide lncRNA expressions in five paired NSCLC tumor and adjacent normal lung tissues were investigated by microarray analysis. Hierarchical cluster analysis of significantly differentially expressed lncRNA: bright red, up‐regulation; bright green, down‐regulation. (B) Left: qRT‐PCR analysis of relative LINC01436 expression in NSCLC tissues (*n* = 100) compared with paired adjacent normal lung tissues (*n* = 100). * Paired *t*‐test, *P *<* *0.05. Right: LINC01436 expression in the TCGA NSCLC dataset (75 normal vs 708 tumor tissues). The LINC01436 expression data were downloaded from the TANRIC website (http://ibl.mdanderson.org/tanric/_design/basic/index.html). The results are expressed as the mean ± SEM. **Mann–Whitney *U*‐test, *P *<* *0.001. (C) Kaplan–Meier curves for OS of NSCLC patients expressing high (*n* = 354) and low (*n* = 354) expression levels of LINC01436. * Log‐rank test, *P *<* *0.05. (D) The coding potential of LINC01436. Coding potentials of lncRNA (LINC01436, MALAT1 and TUG1) and mRNA (GAPDH, ACTB) were calculated using CPAT and CPC. (E) The subcellular localization of LINC01436 by RNA FISH. Blue, DAPI‐stained nuclei; red, Cy3‐labeled positive hybridization signals (scale bar, 20 μm). U6 and 18S were used as positive controls.

We performed a microarray analysis to identify systematically genome‐wide differential lncRNA expression in five paired NSCLC tumor and adjacent normal lung tissues. We identified 332 lncRNA differentially expressed between tumor and paired normal lung tissues (*P* < 0.05 and fold change > 2.0), which consisted of 230 down‐regulated and 102 up‐regulated lncRNA (Fig. 1A, Table S6). LINC01436 was one of the top up‐regulated lncRNA (*P* = 0.029, fold change = 4.33). To validate this result, we investigated the LINC01436 expression levels in 100 paired NSCLC tumor and adjacent normal lung tissues using qRT‐PCR. LINC01436 expression levels were significantly up‐regulated in NSCLC tumor tissues compared with adjacent normal tissues (*P *< 0.05; Fig. 1B). To further confirm our results, we analyzed TCGA data and found that LINC01436 was also significantly up‐regulated in NSCLC tissues (*n* = 708) compared with normal lung tissues (*n* = 75, *P *< 0.01; Fig. 1B).

To assess the clinical significance of LINC01436 in NSCLC, Kaplan–Meier survival analysis was performed to compare the association of LINC01436 expression with patient outcomes using TCGA data. We observed that the high expression of LINC01436 was significantly associated with poor OS (*n* = 708, *P* = 0.044; Fig. 1C). The association with OS in NSCLC patients remained significant using a multivariate Cox proportional hazards regression analysis after adjustment for age, gender, tumor stage, smoking, radiation therapy and targeted molecular therapy (*n* = 708, hazard ratio = 1.495, 95% confidence interval = 1.102–2.208, *P* = 0.010; Table S4). Collectively, these results revealed that LINC01436 was up‐regulated and high expression levels of LINC01436 were associated with poor outcomes in NSCLC.

### 3.2 Characterization of LINC01436

LINC01436 was located at chromosome 21q22.12, consisting of two exons with a full length of 1523 nt (Fig. S1A,B; http://www.ncbi.nlm.nih.gov/RefSeq/). Our 5′‐RACE assays validated the 5′‐transcription start site (TSS) of LINC01436 (Fig. S1C), which was consistent with the annotation of this gene in RefSeq. We next determined whether LINC01436 was a noncoding gene. LINC01436 harbors 11 potential open reading frames (ORF) that might code short peptides of 25–70 amino acids (Fig. S2A) predicted by the ORF Finder (https://www.ncbi.nlm.nih.gov/orffinder/); however, LINC01436 lacks the Kozak sequence, which is important for translation initiation. Moreover, LINC01436 has no coding potential according to a coding potential assessment tool (CPAT) and coding potential calculator (CPC; Fig. 1D). We further used an RNA FISH assay to dissect the subcellular localization of LINC01436. LINC01436 was predominantly observed in cell cytoplasm (Fig. 1E). The qRT‐PCR of nuclear and cytoplasmic fractions confirmed that LINC01436 was mainly located in the cytoplasm (Fig. S2B). Moreover, LINC01436 expression levels were analyzed by qRT‐PCR in five NSCLC cell lines (H1299, 95D, SPCA1, H460 and A549) and a normal human bronchial epithelial cell line (HBE; Fig. S2C). Compared with HBE cell line, H1299 cell line exhibited the highest LINC01436 expression level, and A549 cell line the lowest. Thus, we established a gain‐of‐function cell model by transfecting a LINC01436‐expressing vector into the A549 cell line and knocked down LINC01436 expression in the H1299 cell line in the following study. LINC01436 expression level in SPCA1 cell line was between that of H1299 and A459. Thus, we established gain‐ and loss‐of‐function cell model using SPCA1 cell line.

### 3.3 LINC01436 expression is inhibited by E2F6

**Figure 2:**
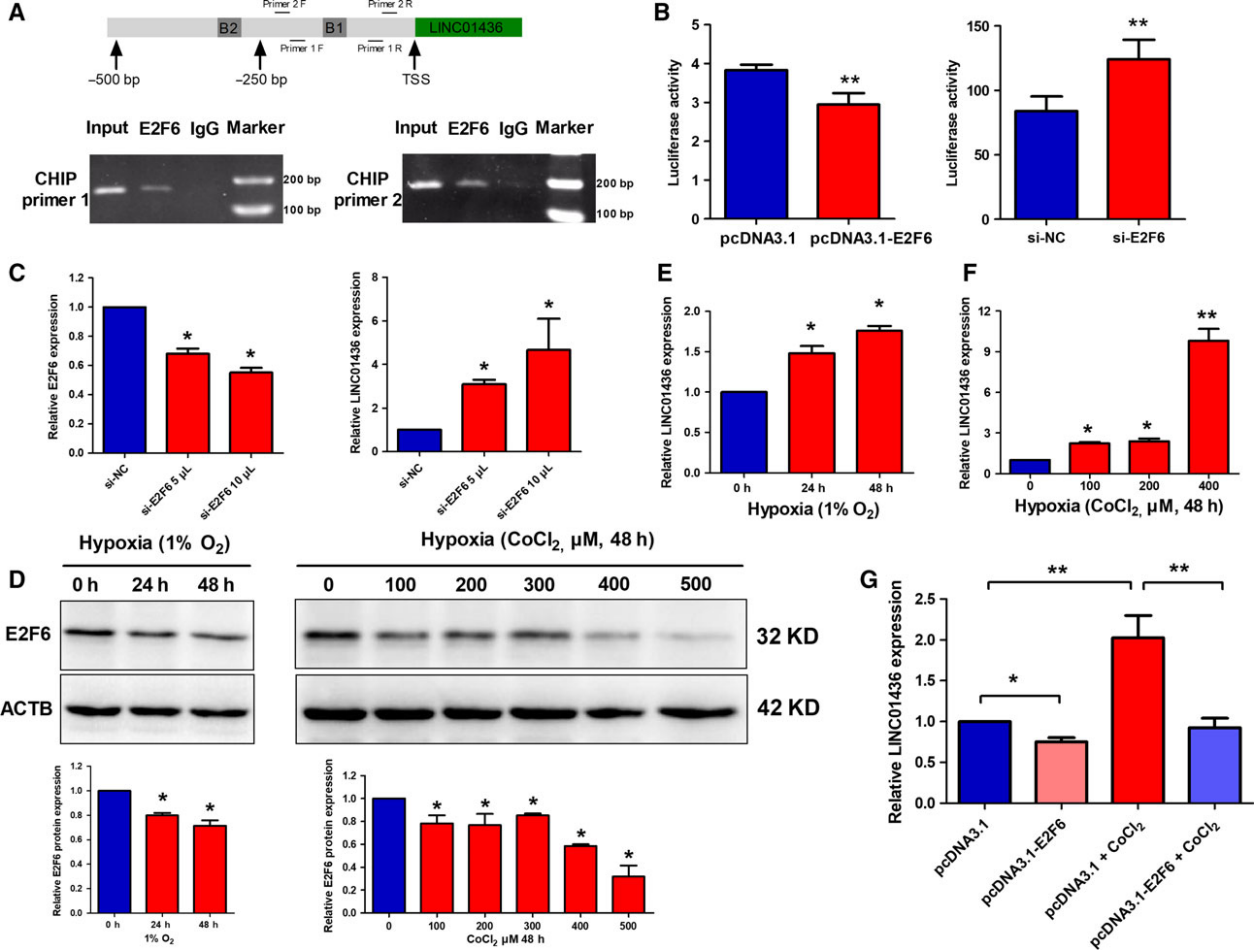
LINC01436 expression is repressed by E2F6 under normoxia and the inhibitory effect is released under hypoxia. (A) Upper chart: Two E2F6 binding sites were predicted in the promoter region (−500 to +100 bp) of LINC01436. B1, binding site 1; B2, binding site 2. Lower chart: ChIP‐PCR assays were performed to detect whether E2F6 directly bound to the promoter of LINC01436. Primers for CHIP‐PCR are also indicated in the schema: F primer, forward primer; R primer, reverse primer. (B) Luciferase assays of H1299 cells co‐transfected with pGL3‐basic‐LINC01436 reporter and E2F6‐overexpression vectors or E2F6 siRNA. The LINC01436 promoter sequence (−354 to +133 bp) was synthesized and subcloned into the pGL3‐basic vector. (C) LINC01436 expression levels in H1299 cells after knockdown of E2F6 by siRNA. (D) Upper chart: E2F6 protein expression levels in H1299 cells cultured in physical hypoxia (1% O_2_) for 24 and 48 h or treated with different doses of CoCl_2_ for 48 h. Lower chart: The quantitative analysis results of three independent WB. (E) LINC01436 expression levels in H1299 cells cultured in physical hypoxia (1% O_2_) for 24 and 48 h. (F) LINC01436 expression levels in H1299 cells treated with different doses of hypoxia‐mimetic agent CoCl_2_ for 48 h. (G) LINC01436 expression levels after E2F6 overexpression under CoCl_2_‐induced hypoxia. Error bars represent the SD of three independent experiments. *Student's *t*‐test, *P *<* *0.05; ** Student's *t*‐test, *P *<* *0.01.

To explore the potential mechanisms controlling the expression of LINC01436, we used the open regulatory annotation (ORegAnno) track in the UCSC genome browser and found an E2F6 binding site (−292 to −281 bp) in the LINC01436 promoter region (Fig. S1A). E2F6 is known as a transcriptional suppressor. A previous study also revealed that E2F6 repressed the oncogenic lncRNA H19 expression in breast cancer cells (Berteaux *et al*., 2005). Thus, we focused on the regulatory effect of E2F6 on LINC01436 expression. We predicted that E2F6 has two specific binding sites (score over 10) in LINC01436 promoter (−500 to +100 bp) using jaspar (http://jaspar.genereg.net/; Figs 2A and S3A). Using ChIP experiments, we confirmed the direct binding of E2F6 to LINC01436 promoter (Fig. 2A). We next performed luciferase reporter assays to validate promoter regulation. The sequences of the LINC01436 promoter region were inserted into the reporter plasmid (Fig. S3A). Co‐transfection of the E2F6‐overexpression vectors with LINC01436 promoter luciferase constructs significantly repressed the luciferase activity, whereas knockdown of E2F6 by siRNA enhanced the activity of LINC01436 promoter (Figs 2B and S3B). We further measured LINC01436 expression in H1299 cells after knockdown of E2F6 by siRNA. Consistent with the results of luciferase reporter assay, knockdown of E2F6 markedly enhanced the expression of LINC01436 compared with the control (Fig. 2C). To avoid the off‐target effect, we tried another siRNA (si‐E2F6‐2) against E2F6. We measured luciferase activity of LINC01436 promoter and LINC01436 expression in H1299 cells after knockdown of E2F6 by si‐E2F6‐2. Consistent with the results of si‐E2F6, knockdown of E2F6 by si‐E2F6‐2 also up‐regulated the luciferase activity and expression of LINC01436 compared with the control (Fig. S4). The above findings indicated that E2F6 repressed the expression of LINC01436 by directly binding to LINC01436 promoter.

### 3.4 LINC01436 expression is up‐regulated under hypoxia

Previous studies have shown that E2F6 expression was down‐regulated under hypoxia in HEK293 cells (Yang *et al*., 2008). We thus wondered whether the inhibitory effect of E2F6 on LINC01436 expression could be relieved under hypoxia. First, we investigated whether E2F6 was affected by hypoxia in lung cancer cells. To this end, H1299 cells were cultured in low oxygen conditions and E2F6 protein levels were decreased in physical hypoxia‐ (1% O_2_) and CoCl_2_‐induced hypoxia (chemically induced pseudo‐hypoxia; Fig. 2D). Then, we investigated the expression levels of LINC01436 under hypoxia. For this purpose, H1299 cells were cultured in the presence of low oxygen tension (1% O_2_; Fig. 2E) or treated with different doses of CoCl_2_ (Fig. 2F). Compared with the non‐hypoxic controls, LINC01436 expression was up‐regulated under hypoxic conditions and displayed a time‐ and dose‐dependent manner. Thus, we can infer that hypoxia‐induced down‐regulation of E2F6 could lead to the up‐regulation of LINC01436. To further confirm this, we examined the expression of LINC01436 after E2F6 overexpression under CoCl_2_‐induced hypoxia. We found that the inhibitory effect of E2F6 on LINC01436 expression is relieved under hypoxia (Fig. 2G).

### 3.5 LINC01436 overexpression promotes proliferation, migration and invasion *in vitro*

**Figure 3:**
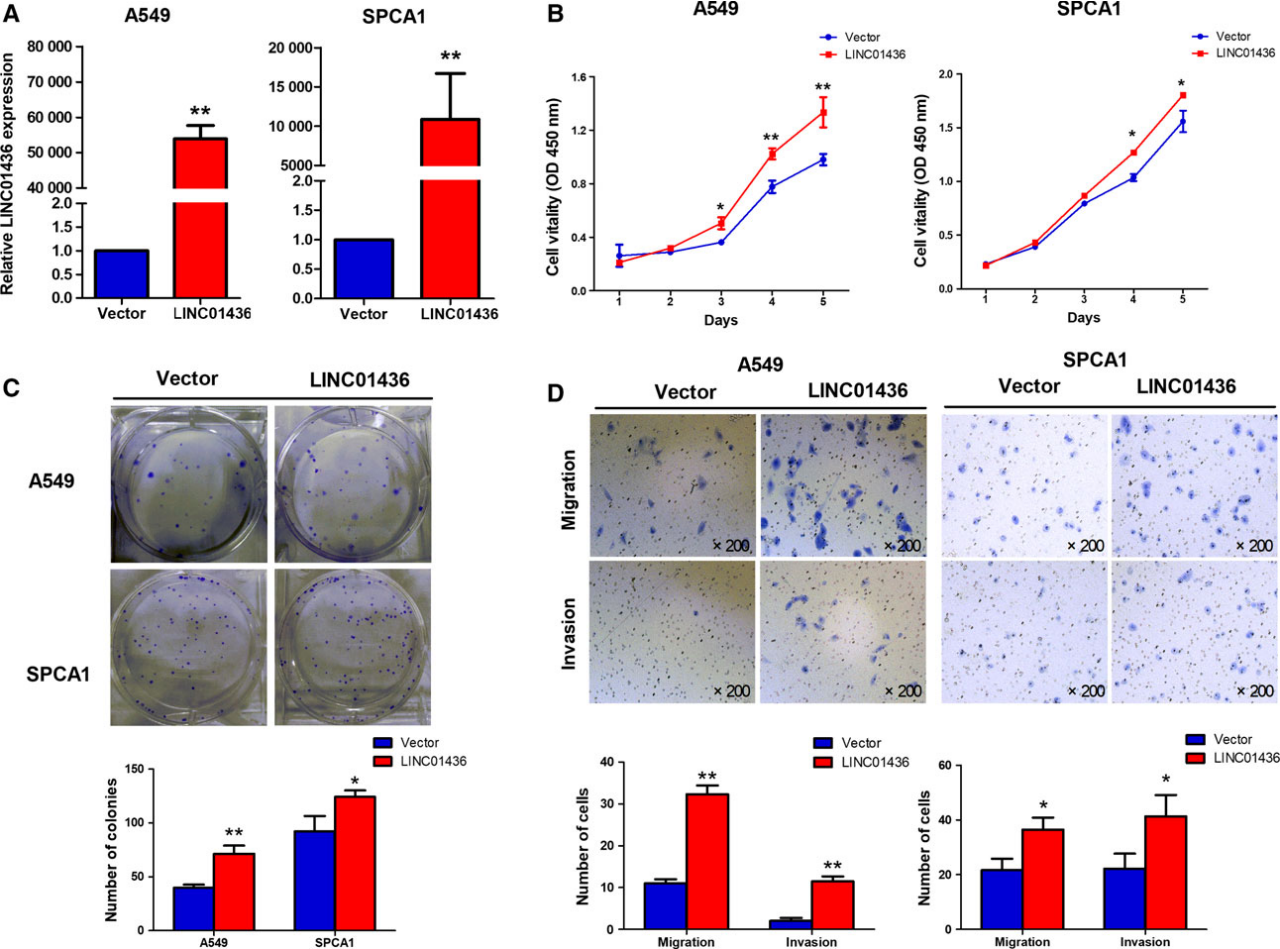
Overexpression of LINC01436 promotes cell proliferation, migration and invasion *in vitro*. (A) Overexpression of exogenous LINC01436 in A549 and SPCA1 cells were identified by qRT‐PCR. (B) Growth curves of A549 and SPCA1 cells after transfection with LINC01436 or vector control were examined by CCK‐8 assays. (C) The effect of LINC01436 on cell proliferation was further determined by a colony formation assay. Visible colonies were counted. (D) Migration and invasion of A549 and SPCA1 cells transfection with LINC01436 or vector control were determined by transwell assays. Magnification, ×200. Error bars represent the SD of three independent experiments. *LINC01436 vs vector control, Student's *t*‐test, *P *<* *0.05; * LINC01436 vs vector control, Student's *t*‐test, *P *<* *0.01.

To evaluate the potential role of LINC01436 in NSCLC, we established gain‐of‐function cell models by transfecting pcDNA3.1‐LINC01436 expressing vectors into the A549 and SPCA1 cells (Fig. 3A). We examined the effects of LINC01436 overexpression on cell proliferation, migration and invasion. CCK‐8 assays showed that overexpression of LINC01436 significantly promoted the proliferation of A549 and SPCA1 cells (Fig. 3B). The proliferation promotion effect of LINC01436 was confirmed by colony formation assays, which showed that LINC01436 overexpression could boost the number of A549 and SPCA1 clones (Fig. 3C). Moreover, the *in vitro* transwell assays showed that overexpression of LINC01436 significantly increased the migration and invasion of A549 and SPCA1 cells compared with vector control (Fig. 3D).

### 3.6 Knockdown of LINC01436 inhibits proliferation, migration and invasion *in vitro*

**Figure 4:**
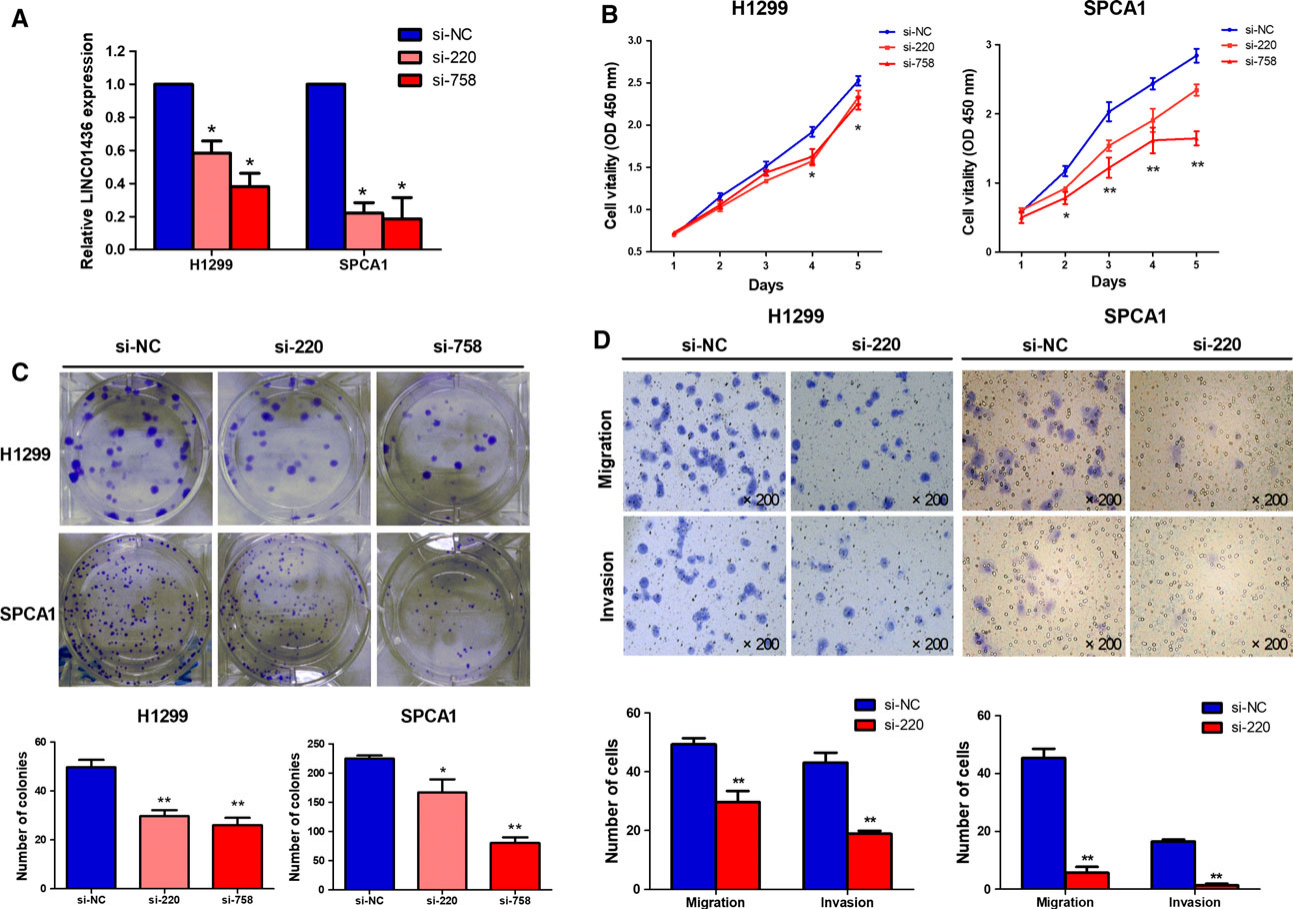
Knockdown of LINC01436 inhibits cell proliferation, migration and invasion *in vitro*. (A) Knockdown of LINC01436 expression in H1299 and SPCA1 cells was identified by qRT‐PCR. (B) Growth curves of H1299 and SPCA1 cells after transfection with LINC01436 siRNAs or negative control si‐NC were examined by CCK‐8 assays. (C) The effects of LINC01436 knockdown on cell proliferation were further confirmed by a colony formation assay. Visible colonies were counted. (D) Migration and invasion of H1299 and SPCA1 cells transfection with si‐220 or si‐NC were determined by transwell assays. Magnification, ×200. Error bars represent the SD of three independent experiments. *si‐220 or si‐758 vs si‐NC, Student's *t*‐test, *P *<* *0.05; **LINC01436 vs vector control, Student's *t*‐test, *P *<* *0.01.

We knocked down the expression of LINC01436 in H1299 and SPCA1 cells using two independent siRNA. LINC01436 expression levels were significantly reduced after siRNA transfection (Fig. 4A). In contrast to the gain‐of‐function cell models, knockdown of LINC01436 significantly decreased H1299 and SPCA1 cell proliferation (Fig. 4B) and clonogenic survival (Fig. 4C). Moreover, the *in vitro* transwell assays showed that LINC01436 knockdown significantly impeded the migration and invasion of H1299 and SPCA1 cells (Fig. 4D).

### 3.7 LINC01436 promotes tumor growth and metastasis *in vivo*

**Figure 5:**
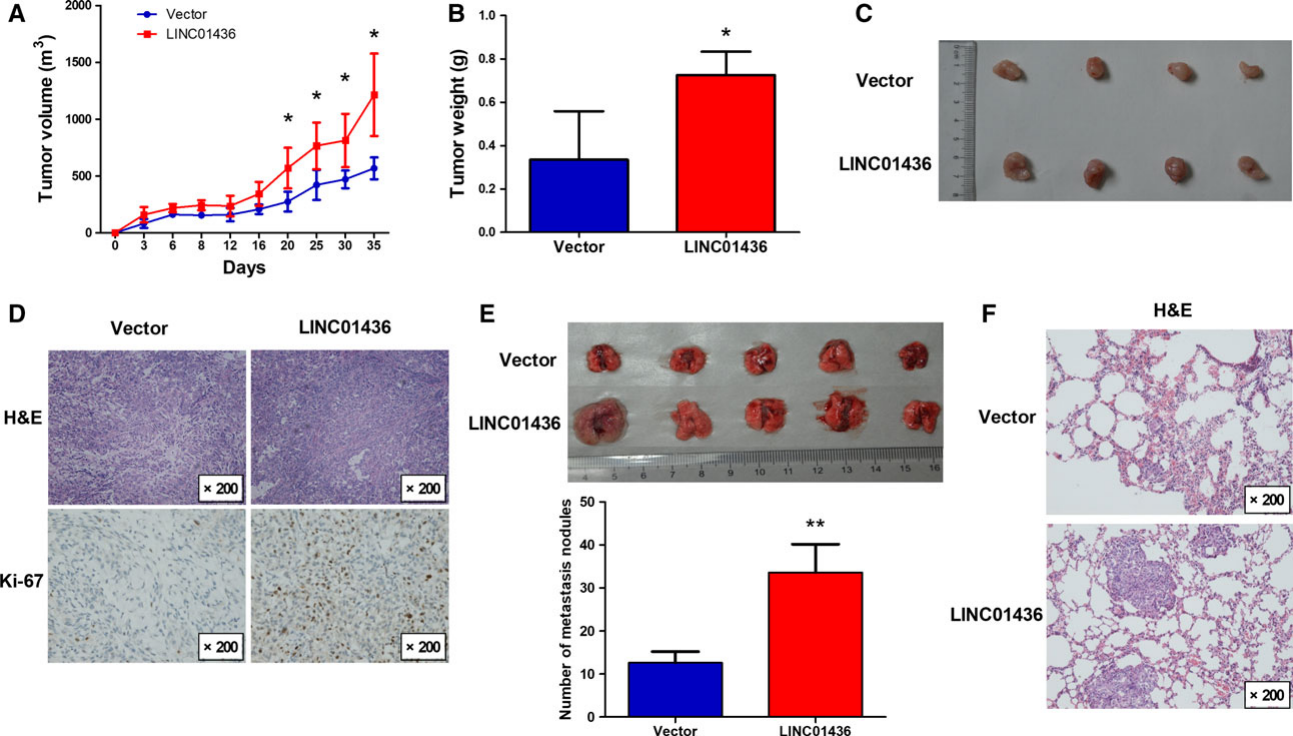
Overexpression of LINC01436 promotes lung cancer cell growth and metastasis *in vivo*. (A) The tumor growth curve of A549 cells stable overexpressing LINC01436 or control vector. Stable transfected A549 cells (5.0 × 10^6^) were subcutaneously injected into the left flank of nude mice. The tumor volume was calculated using the equation *V* = 0.5 × *D* × *d* ^2^ where *V =* volume; *D* = longitudinal diameter and *d =* latitudinal diameter. Error bars represent the SD of four independent experiments. (B) Tumor weight from the LINC01436 and vector control groups. Error bars represent the SD of four independent experiments. (C) Tumors from the LINC01436 and vector control groups upon resection from BALB/c‐nude mice. (D) Immunohistochemical analysis of Ki‐67 was performed to assess tumor cell proliferation. Magnification, ×200. (E) Lungs from nude mice in each group 5 weeks after injections of stable transfected A549 cells (1.0 × 10^6^). The number of lung metastatic nodules on lung surfaces was counted. Error bars represent the SD of five independent experiments. (F) H&E staining of lung tissue slices confirmed that more metastatic nodules were present in LINC01436 group than in the vector control group. Magnification, ×200. *LINC01436 vs vector control, Student's *t*‐test, *P *<* *0.05; **LINC01436 vs vector control, Student's *t*‐test, *P *<* *0.01.

To further determine whether LINC01436 promotes tumorigenesis *in vivo*, we used subcutaneous xenograft tumor models to assess the growth of A549 cells stably transfected with LINC01436 or control vector in nude mice. The tumor volume and weight in the LINC01436 overexpression group were significantly larger and heavier than those in control group (Fig. 5A,B,C). Further, proliferation marker Ki‐67 was evaluated through immunohistochemistry in tumor tissues. Ki‐67 expression levels were significantly higher in the LINC01436 overexpression group than in the control vector group (Fig. 5D).

To validate the pro‐metastatic effects of LINC01436 *in vivo*, A549 cells stably transfected with LINC01436 or control vector were injected into the tail veins of nude mice. The number of metastatic nodules on the surface of the lung was significantly increased in mice receiving LINC01436 stable overexpressing A549 cells compared with the control (Fig. 5E). This difference was further confirmed by the examination of the lungs by hematoxylin and eosin (H&E) staining of the mice lung sections (Fig. 5F). Collectively, our *in vivo* and *in vitro* data supported LINC01436 as an oncogenic lncRNA in NSCLC.

### 3.8 LINC01436 serves as a sponge for miR‐30a‐3p

**Figure 6:**
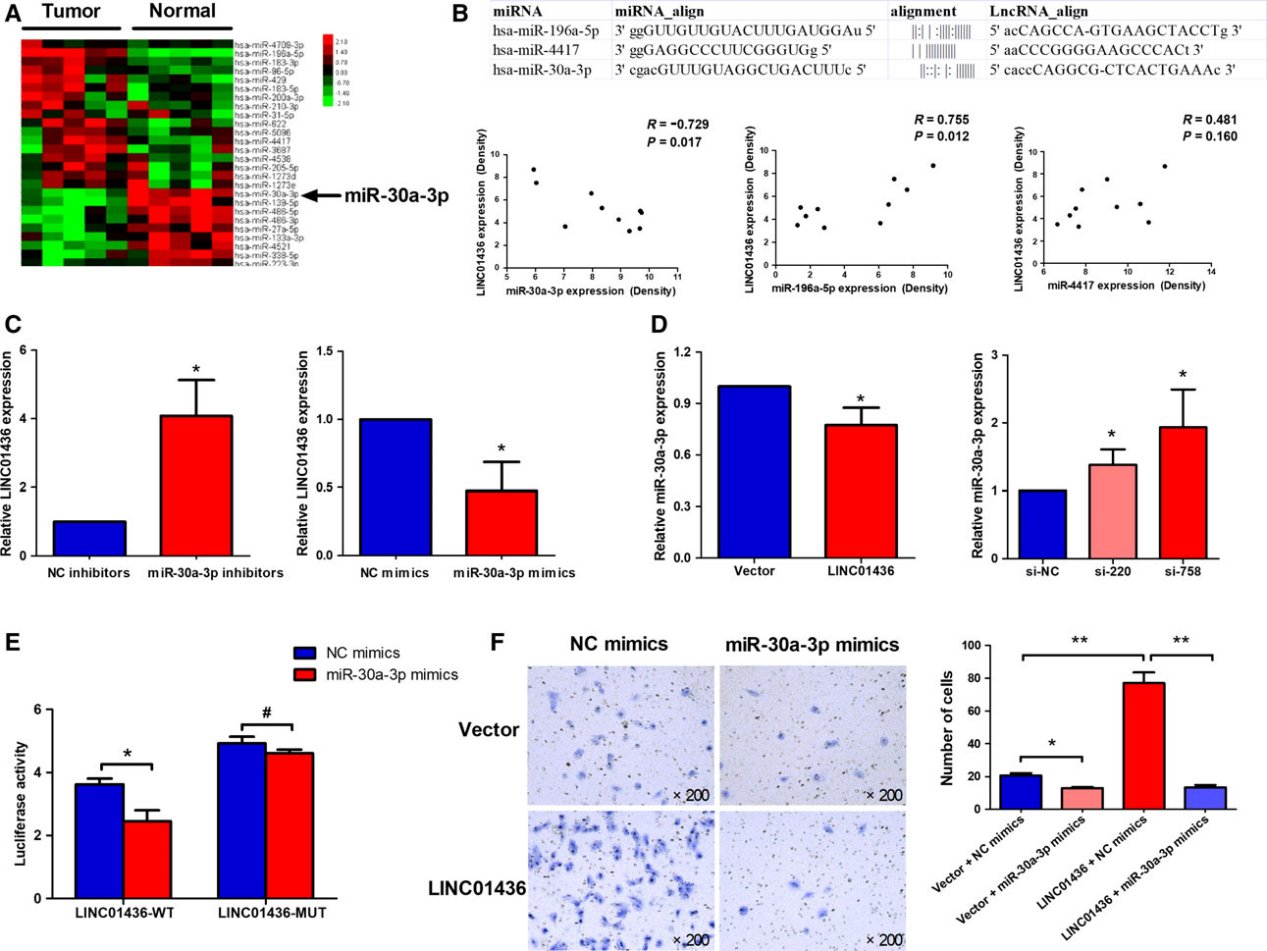
LINC01436 functions as a sponge for miR‐30a‐3p and exerted its function through miR‐30a‐3p. (A) Genome‐wide miRNA expression in the same five paired lung tumor tissues and adjacent normal lung tissues were investigated by microarray analysis. Hierarchical cluster analysis of significantly differentially expressed miRNA: bright red, up‐regulation; bright green, down‐regulation. (B) Potential miRNA were predicted to target LINC01436 by the miRanda algorithm (upper). A Pearson correlation was performed to analyze the correlations between LINC01436 and three potential target miRNA (lower). (C) LINC01436 expression levels in A549 cells after transfection with miR‐30a‐3p inhibitors or miR‐30a‐3p mimics. (D) MiR‐30a‐3p expression levels were detected in A549 cells after overexpression or knockdown of LINC01436. (E) Luciferase assays of A549 cells co‐transfected with LINC01436‐WT or LINC01436‐MUT reporter and miR‐30a‐3p mimics or negative control (NC) mimics. (F) Migration of A549 cells after co‐transfection with LINC01436 or control vector and miR‐30a‐3p mimics or NC mimics. Magnification, ×200. Error bars represent the SD of three independent experiments. *Student's *t*‐test, *P *<* *0.05; **Student's *t*‐test, *P *<* *0.01; ^#^Student's *t*‐test, *P *>* *0.05.

We next investigated the biological mechanism by which LINC01436 contributed to lung cancer progression. Since LINC01436 was distributed predominantly in cell cytoplasm (Fig. 1E), we hypothesized LINC01436 might function as natural miRNA sponges to prevent miRNA from binding their target mRNA. We first explored differentially expressed miRNA in the abovementioned five paired tissues by microarray. According to miRNA profiles, 26 miRNA was found to be significantly differentially expressed between the tumor tissues and matched normal lung tissues, 17 up‐regulated and nine down‐regulated (Fig. 6A, Table S7). We validated our results with TCGA data and found that the expression patterns of miRNA in TCGA data were consistent with our microarray data (Table S5). Among the deregulated miRNA, miR‐30a‐3p, miR‐196a‐5p and miR‐4417 were predicted to target LINC01436 using the miRanda algorithm (Fig. 6B). Further analysis showed that only miR‐30a‐3p was significantly negatively correlated with LINC01436 expression (*r* = −0.729, *P* = 0.017; Fig. 6B), suggesting that LINC01436 may serve as a sponge for miR‐30a‐3p in lung cancer. Subsequently, we investigated the expression levels of LINC01436 after knockdown or overexpression of miR‐30a‐3p. The miR‐30a‐3p inhibitors significantly increased LINC01436 expression levels, whereas the miR‐30a‐3p mimicked generated significant down‐regulation of LINC01436 in A549 cells compared with control (Fig. 6C). In contrast, after overexpression or knockdown of LINC01436 in A549 cells, miR‐30a‐3p was down‐regulated or up‐regulated, respectively (Fig. 6D). For further confirmation, the sequences of LINC01436, which contains wild‐type or mutated miR‐30a‐3p binding sites (LINC01436‐MUT), were inserted into the pMIR‐RB‐REPORT™ luciferase vectors (Fig. S5A). Overexpression of miR‐30a‐3p significantly reduced the luciferase activities of the LINC01436‐WT reporter vector but not the LINC01436‐MUT reporter vector (Fig. 6E). Functionally, miR‐30a‐3p could inhibit the migration of A549 cells, and overexpression of miR‐30a‐3p rescued the migration‐promoting effect of LINC01436 in A549 cells (Figs 6F and S5B). These results implied that LINC01436 could function as a natural sponge for miR‐30a‐3p and that the oncogenic effects of LINC01436 were dependent on miR‐30a‐3p.

### 3.9 LINC01436 regulates the expression of the miR‐30a‐3p target gene, EPAS1

**Figure 7:**
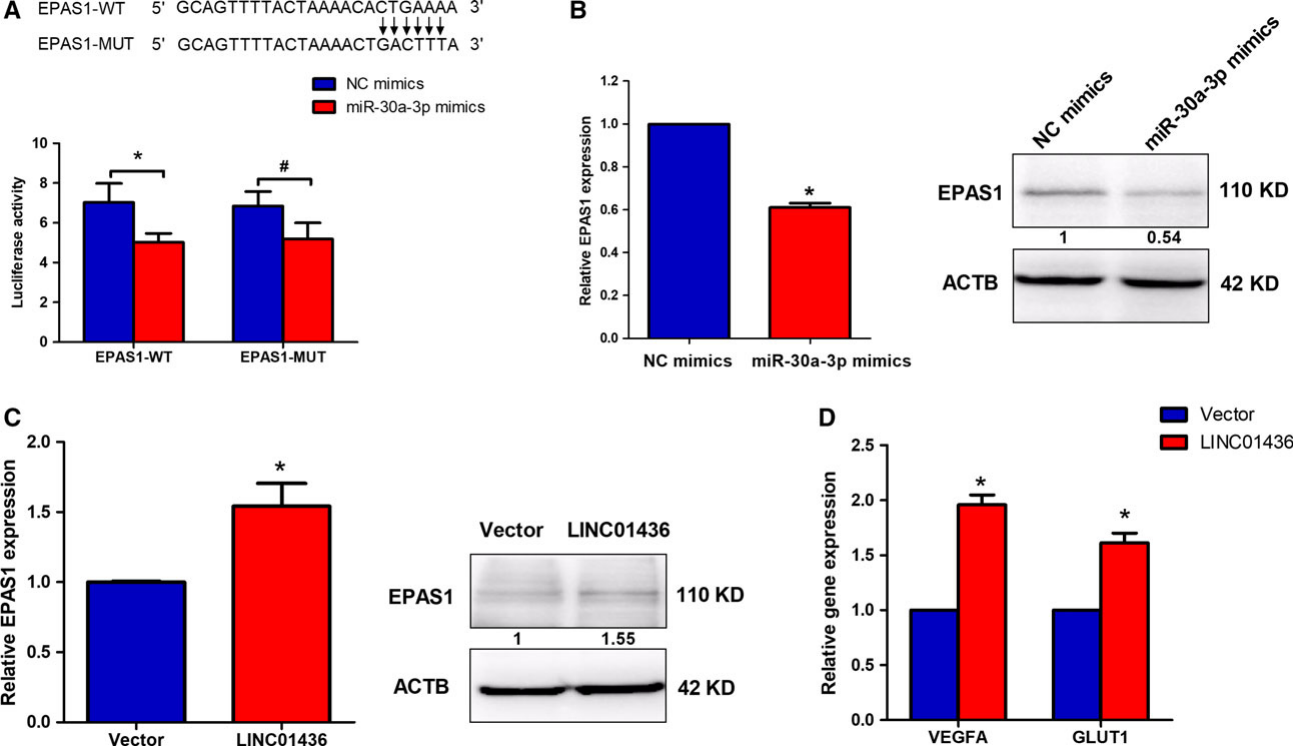
EPAS1 is a target gene of LINC01436 through miR‐30a‐3p. (A) Luciferase assays of A549 cells co‐transfected with EPAS1‐WT or EPAS1‐MUT reporter and miR‐30a‐3p mimics or NC mimics. (B) EPAS1 mRNA and protein expression levels in A549 cells were detected by qRT‐PCR and WB after transfection with miR‐30a‐3p mimics or NC mimics. The numbers in the figure indicate the quantitative analysis results. (C) EPAS1 mRNA and protein expression levels in A549 cells were detected by qRT‐PCR and WB after overexpression of LINC01436. The numbers in the figure indicate the quantitative analysis results. (D) EPAS1 target gene expression levels in A549 cells were detected by qRT‐PCR after overexpression of LINC01436. Error bars represent the SD of three independent experiments. *Student's *t*‐test, *P *<* *0.05; ^#^Student's *t*‐test, *P *>* *0.05.

Seven cancer‐related genes, including *EPAS1*,* CREBBP*,* MECP2*,* THBS2*,* GALNT7*,* ABCA1* and *TNFSF13B*, were predicted by miRanda to be miR‐30a‐3p target genes (Fig. S6A). Therefore, we investigated which of the seven putative targets of miR‐30a‐3p were down‐regulated after LINC01436 knockdown. We found that only *EPAS1* mRNA was down‐regulated after LINC01436 knockdown (Fig. S6B). To verify that *EPAS1* was a target of miR‐30a‐3p, the sequences of wild‐type *EPAS1* mRNA 3′UTR with the miR‐30a‐3p binding site, and the mutations (*EPAS1* mRNA 3′UTR MUT), were inserted into the pMIR‐RB‐REPORT™ luciferase vectors (Fig. 7A). MiR‐30a‐3p overexpression significantly reduced the luciferase activities of the EPAS1‐WT reporter vector but not the EPAS1‐MUT reporter vector (Fig. 7A). Moreover, qRT‐PCR and WB analysis showed that EPAS1 was negatively regulated by miR‐30a‐3p in A549 cells (Fig. 7B). These results indicated that EPAS1 was a direct target of miR‐30a‐3p. Furthermore, the qRT‐PCR and WB analysis showed ectopic overexpression of LINC01436 increased EPAS1 mRNA and protein expression levels in A549 cells (Fig. 7C). Ectopic overexpression of LINC01436 also increased EPAS1 target genes, including vascular endothelial growth factor A (*VEGFA*) and glucose transporter 1 (*GLUT1*) in A549 cells (Figs 7D and S7).

## 4 Discussion

In this study, we demonstrated that LINC01436, a long intergenic noncoding RNA, was significantly up‐regulated in NSCLC tumor tissues and the high expression of LINC01436 was associated with poor outcomes in NSCLC. Functional studies revealed that LINC01436 promoted lung cancer cells proliferation, migration and invasion *in vitro* and promoted tumor growth and metastasis *in vivo*.

The subcellular localization patterns have gradually been proved to be a key feature for deep understanding of lncRNA biological function (Chen, 2016). By using an RNA FISH assay and cell cytoplasm/nucleus fraction isolation, we found LINC01436 was preferentially localized in the cytoplasm. LncRNA in the cytoplasm could provide a multi‐hued palette of regulatory capacities at posttranscriptional level, such as impact on the half‐life or translation potential of mRNA (Batista and Chang, 2013). Recently, emerging studies pointed out that cytoplasmic lncRNA could function as ‘miRNA sponges’ to inhibit miRNA function with high efficiency and specificity (Li *et al*., 2017; Salmena *et al*., 2011; Song and Yin, 2016; Sun *et al*., 2017; Xie *et al*., 2017). To clarify whether LINC01436 could function as miRNA sponges, we started with microarray profiling and TCGA data to detect differentially expressed miRNA in NSCLC. Then, we performed bioinformatics analysis to find the potential miRNA binding sites of LINC01436 among the deregulated miRNA. Based on a correlation analysis of microarray data, we focused on the potential regulation network between LINC01436 and miR‐30a‐3p. Subsequent qRT‐PCR and dual‐luciferase reporter assays confirmed that LINC01436 was a direct target of miR‐30a‐3p. Our data supported that LINC01436 functions as an endogenous sponge for miR‐30a‐3p.

It has been proved that miR‐30a‐3p was down‐regulated in several cancers: breast cancer (Perez‐Rivas *et al*., 2014), endometrioid endometrial carcinoma (Tsukamoto *et al*., 2014) and papillary thyroid carcinoma (Peng *et al*., 2014). Functional studies also revealed miR‐30a‐3p could act as a tumor suppressor in hepatocellular carcinoma (Wang *et al*., 2014), clear cell renal cell carcinomas (Mathew *et al*., 2014) and gastric cancer (Liu *et al*., 2017). Very recently, several studies highlighted the role of miR‐30a‐3p in lung cancer, and demonstrated that miR‐30a‐3p was significantly decreased in lung cancer and that miR‐30a‐3p down‐regulation played important roles in the metastatic progression of lung cancer (Daugaard *et al*., 2017; Xie *et al*., 2016; Zhang *et al*., 2017). More importantly, tumor‐derived exosomal miR‐30a‐3p was specifically down‐regulated in NSCLC patients and could serve as diagnostic biomarkers for early‐stage NSCLC (Jin *et al*., 2017). In our study, microarray analysis and TCGA data both showed that miR‐30a‐3p was down‐regulated in NSCLC. The tumor‐suppressive effects of miR‐30a‐3p were also observed *in vitro*. Additionally, rescue experiments demonstrated that miR‐30a‐3p restored the tumor‐promoting effects of LINC01436, indicating that LINC01436 exerted its function through miR‐30a‐3p in NSCLC.

Recent studies have identified *EPAS1* (Mathew *et al*., 2014), *CREBBP* (Kumarswamy *et al*., 2014), *MECP2* (Volkmann *et al*., 2013) and *TNFSF13B* (Alsaleh *et al*., 2014) as target genes of miR‐30a‐3p. Our study showed LINC01436 acts as a ceRNA to regulate EPAS1 by sponging miR‐30a‐3p in lung cancer. *EPAS1*, also named *HIF‐2*α, was associated with therapy resistance, metastasis and poor clinical prognosis in various cancers, including lung cancer (Ma *et al*., 2016; Zhao *et al*., 2015). High EPAS1 protein levels were detected in ~ 50% of cases and were strongly correlated with poor OS in NSCLC (Giatromanolaki *et al*., 2001; Wu *et al*., 2011), suggesting that EPAS1 may directly contribute to NSCLC progression. Ectopic overexpression of non‐degradable EPAS1 proteins cooperated with RAS to promote growth of Kras^G12D^‐driven murine lung tumors (Kim *et al*., 2009). These studies suggested that increased EPAS1 activity plays important roles in lung cancer. However, the expression and clinical significance of EPAS1 in NSCLC tissues has not been definitively established. A recent multi‐omics analysis revealed that lung tumor tissues had low EPAS1 mRNA and protein expression compared with normal tissues (Wang *et al*., 2018). In our present study, *EPAS1* mRNA expression levels were not correlated with LINC01436 expression levels in lung tumor tissues and no difference was found for EPAS1 protein expression between lung tumor tissues and adjacent normal lung tissues (Fig. S8). Thus, additional well‐designed clinical studies are warranted to better understand the expression and clinical significance of EPAS1 in NSCLC tissues.

Here, we demonstrated that LINC01436 regulated the expression of EPAS1 and its target genes in lung cancer cell lines, including angiogenesis (*VEGFA*) and metabolism reprogramming (*GLUT1*) genes (Raval *et al*., 2005; Wiesener *et al*., 1998). These genes were involved in critical steps of phenotype changes in cancer progression, underlining the impact of LINC01436 in lung tumorigenesis. Moreover, EPAS1 protein was found to be sensitive to hypoxia and was primarily degraded under normoxia via the von Hippel‐Lindau protein (pVHL) recognition, polyubiquitination and proteasomal degradation (Kim and Kaelin, 2003). In addition to the canonical posttranslational degradation mechanism, we showed here that *EASP1* mRNA and protein expression could also be modulated by lncRNA through a ceRNA mechanism. This new mode of HIF‐α regulation has also been found in HIF‐1α expression. The expression of HIF‐1α was modulated by lncRNA linc‐RoR functioning as an miR‐145 sponge (Takahashi *et al*., 2014; Wang *et al*., 2013). Interestingly, the expression of linc‐RoR itself could also be induced by hypoxia (Takahashi *et al*., 2014).

**Figure 8:**
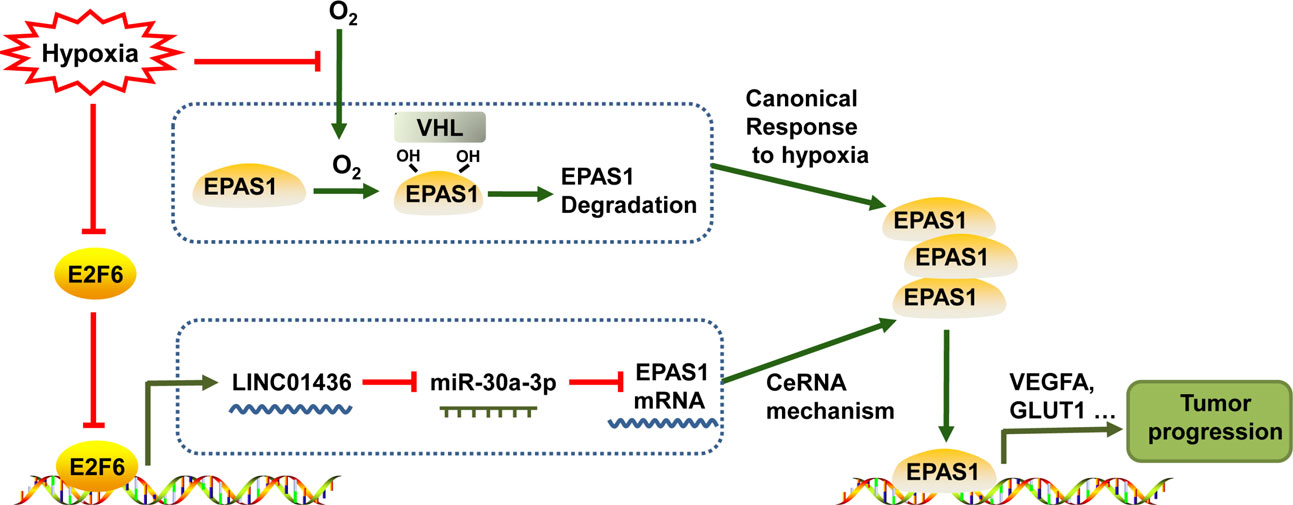
A proposed model depicting the potential mechanisms of LINC01436 as an oncogenic lncRNA in NSCLC. Under hypoxia, E2F6 protein is down‐regulated, which leads to the up‐regulation of LINC01436. Up‐regulated LINC01436 stimulates the expression of EPAS1 through miR‐30a‐3p. EPAS1 mediates the cellular response to hypoxia and regulates target genes critical for tumor progression, such as *VEGFA* (angiogenesis) and *GLUT*1 (metabolism).

Hypoxia is a common phenomenon during various types of cancer progression (Gilkes *et al*., 2014; Rankin and Giaccia, 2016). Several hypoxia‐sensitive lncRNA have been involved in tumorigenesis (Shih and Kung, 2017). For instance, hypoxia can increase the expression of lincRNA‐p21 (Yang *et al*., 2014), MIR31HG (Shih *et al*., 2017), linc‐ROR (Takahashi *et al*., 2014) and aHIF (Thrash‐Bingham and Tartof, 1999), and repress the expression of lncRNA‐LET (Yang *et al*., 2013). In our study, we demonstrated LINC01436 as another hypoxia‐sensitive lncRNA. Using the UCSC genome browser and jaspar, we predicted two potential E2F6 binding sites in LINC01436 promoter. Using a CHIP experiment, we confirmed that the promoter of LINC01436 was occupied by the transcriptional repressor E2F6, which mediated the repression of LINC01436. Interestingly, repression of E2F6 by hypoxia was also observed in lung cancer cells, and the inhibitory effect of E2F6 on LINC01436 expression was relieved under hypoxia. Thus, we propose that hypoxia‐sensitive LINC01436 regulates EPAS1 expression by functioning as a ceRNA. In a hypoxic microenvironment, E2F6 protein is down‐regulated, which leads to up‐regulation of LINC01436 expression. In contrast to the canonical response to hypoxia, the expression of EASP1 is also stimulated by up‐regulated LINC01436 through miR‐30a‐3p. EPAS1 activation, ultimately, mediates the cellular response to hypoxia and regulates target genes critical for tumor progression, such as *VEGFA* (angiogenesis) and *GLUT1* (metabolism; Fig. 8).

## 5 Conclusions

This study demonstrates that LINC01436 is up‐regulated and acts as an oncogene in NSCLC. The high expression of LINC01436 is an independent risk factor for OS in patients with NSCLC. LINC01436 is repressed by E2F6 under normoxia, whereas its inhibitory effect is relieved under hypoxia. Moreover, hypoxia‐induced abnormal high expression of LINC01436 stimulates EPAS1 expression by functioning as a ceRNA. Our results suggest that LINC01436 could play important roles in hypoxia‐regulated cancer progression, and may be a potential biomarker in prognosis and a therapeutic target for NSCLC.

## Conflict of interest

The authors declare no conflict of interest.

## Author contributions

Y.L. led the study by designing, conducting, interpreting results, writing the manuscript, and obtaining the funding. S.Y. performed the majority of the experiments and participated in study design, result interpretation and manuscript writing. Y.X. performed the experiments and coordinated result interpretation and manuscript writing. L.B. and Z.Y. participated in study design, participant recruitment, result interpretation and funding support. G.W., M.Z. and Z.H. performed plasmid construction, cell transfection and animal experiments. Q.L. and C.L. collected human tissue samples and clinical data. G.M. undertook pathology verification. W.X. performed the bioinformatics analysis. N.W. and L.W. participated in data collection and laboratory work. T.C., X.M. and Y.Z. contributed to interpretation of the results and discussions. All authors contributed to the final paper.

## Supporting information


**Fig. S1.** Genomic location and the full length of LINC01436.
**Fig. S2.** Potential ORF and subcellular localization of LINC01436.
**Fig. S3.** LINC01436 expression is repressed by E2F6.
**Fig. S4.** The luciferase activity of LINC01436 promoter and LINC01436 expression in H1299 cells after knockdown of E2F6 by si‐E2F6‐2.
**Fig. S5.** LINC01436 serves as a sponge for miR‐30a‐3p and the tumor‐suppressive effects of miR‐30a‐3p in lung cancer cells.
**Fig. S6.** EPAS1 is a target gene of LINC01436 through miR‐30a‐3p.
**Fig. S7.** Immunohistochemical analysis was performed to assess the protein expression levels of EPAS1 target genes (*VEGFA* and *GLUT1*).
**Fig. S8.** Immunohistochemical analysis was performed to assess the protein expression levels of EPAS1.
**Table S1.** Clinical characteristics of five NSCLC patients for microarray analysis
**Table S2.** Clinical characteristics of 100 NSCLC patients for qRT‐PCR analysis
**Table S3.** Primers and RNA oligonucleotide sequences used in this study.
**Table S4.** Univariate and multivariate Cox regression analyses of OS in NSCLC patients
**Table S5.** MicroRNA expression validation by TCGA datasets

**Table S6.** Profile of differentially expressed lncRNA.

**Table S7.** Profile of differentially expressed microRNA.
