# Supplementary material for: Hypoxia‐sensitive LINC01436 is regulated by E2F6 and acts as an oncogene by targeting miR‐30a‐3p in non‐small cell lung cancer
Source: Mol Oncol. 2019 Jan 30;13(4):840–56. doi: 10.1002/1878-0261.12437 (PMC6441908; doi:10.1002/1878-0261.12437)
Supplement: Supplementary file 1 — Fig. S1. Genomic location and the full length of LINC01436. Fig. S2. Potential ORF and subcellular localization of LINC01436. Fig. S3. LINC01436 expression is repressed by E2F6. Fig. S4. The luciferase activity of LINC01436 promoter and LINC01436 expression in H1299 cells after knockdown of E2F6 by si‐E2F6‐2. Fig. S5. LINC01436 serves as a sponge for miR‐30a‐3p and the tumor‐suppressive effects of miR‐30a‐3p in lung cancer cells. Fig. S6. EPAS1 is a target gene of LINC01436 through miR‐30a‐3p. Fig. S7. Immunohistochemical analysis was performed to assess the protein expression levels of EPAS1 target genes (VEGFA and GLUT1). Fig. S8. Immunohistochemical analysis was performed to assess the protein expression levels of EPAS1. Table S1. Clinical characteristics of five NSCLC patients for microarray analysis Table S2. Clinical characteristics of 100 NSCLC patients for qRT‐PCR analysis Table S3. Primers and RNA oligonucleotide sequences used in this study. Table S4. Univariate and multivariate Cox regression analyses of OS in NSCLC patients Table S5. MicroRNA expression validation by TCGA datasets [file MOL2-13-840-s001.doc]

**Supplementary Table S1. Clinical characteristics of 5 NSCLC patients for microarray analysis**

| **Patient No.** | **Gender** | **Age** | **Tumor purity (%)** | **Tumor**  **grade** | **Tumor stage** |
| --- | --- | --- | --- | --- | --- |
| 1 | Female | 57 | 90 | Poorly differentiated | IIa |
| 2 | Female | 56 | 80 | Moderately differentiated | Ib |
| 3 | Female | 60 | 70 | Moderately differentiated | Ia |
| 4 | Female | 57 | 80 | Moderately differentiated | IIa |
| 5 | Female | 57 | 70 | Moderately differentiated | IIa |

**Supplementary Table S2. Clinical characteristics of 100 NSCLC patients for qRT-PCR analysis**

| **Patient characteristics** | **NSCLC**  **n=100** |
| --- | --- |
| **Age at diagnosis (years), Mean (SD)** | 56.53 (11.37) |
| **Histological type (%)** |  |
| Adenocarcinoma | 72 (72.0) |
| Squamous cell carcinoma | 28(28.0) |
| **Gender (%)** |  |
| Male | 61 (61.0) |
| Female | 39 (39.0) |
| **Tumor stage (%)** |  |
| I | 50 (50.0) |
| II | 33 (33.0) |
| III | 13 (13.0) |
| IV | 3 (3.0) |
| Not available | 1 (1.0) |
| **Lymph nodes metastasis (%)** |  |
| Absent | 71 (71.0) |
| Present | 29 (29.0) |
| **Smoking history (%)** |  |
| Never smoked | 37 (37.0) |
| Smoker | 49 (49.0) |
| Not available | 14 (14.0) |

**Supplementary Table S3. Primers** and RNA oligonucleotides sequences used in this study.

| **Gene** | **Sequence (5’-3’)** | |
| --- | --- | --- |
| **LINC01436** | Forward: | AGATGCCAGGGGTAAAGTGG |
|  | Reverse: | GCATGGGTTCTAGAGTGGGC |
| **EPAS1** | Forward: | TGGTAGCCCTCTCCAACAAG |
|  | Reverse: | CTTCATCCGTTTCCACATCA |
| **VEGFA** | Forward: | CCCACTGAGGAGTCCAACAT |
|  | Reverse: | TTTCTTGCGCTTTCGTTTTT |
| **GLUT1** | Forward: | GGCCAAGAGTGTGCTAAAGAA |
|  | Reverse: | ACAGCGTTGATGCCAGACAG |
| **E2F6** | Forward: | GACCTCGTTTTGATGTATCGCTG |
|  | Reverse: | ATACACTCTCCGCTTTCGGAC |
| ***β*-actin** | Forward: | CCACGAAACTACCTTCAACTCC |
|  | Reverse: | GTGATCTCCTTCTGCATCCTGT |
| **miR-30a-3p** | RT primer: | GTCGTATCCAGTGCAGGGTCCGAGGTATTCGCACTGGATACGACGCTGCT |
|  | Forward: | AAGGCGGCTTTCAGTCGGATGTT |
|  | Reverse: | ATCCAGTGCAGGGTCCGAGG |
| **U6** | Forward: | CTCGCTTCGGCAGCACA |
|  | Reverse: | AACGCTTCACGAATTTGCGT |
| **LINC01436-1 (CHIP-PCR)** | Forward: | TATGTGAATAACTCCTCCTCTC |
|  | Reverse: | AGTCTGATGTAACTGCTACG |
| **LINC01436-2 (CHIP-PCR)** | Forward: | GTCCGTTGGTGGTATGTG |
|  | Reverse: | ACAGGAAGTGTCTCAGTCT |
| **LINC01436 si-220**  **(double strand)** | Sense: | CCAACGUGAGAUUCUCCUUTT |
| **LINC01436 si-758**  **(double strand)** | Sense: | GCCAGUGAAGCUACCUGAUTT |
| **si-E2F6 (double strand)** | Sense: | GGAAGUUACCCAGUCTCCUTT |
| **si-E2F6-2 (double strand)** | Sense: | AGGAGACUGGGUAACUUCCTT |
| **miR-30a-3p mimics** (**single strand)** | Sense: | CUUUCAGUCGGAUGUUUGCAGC |
| **miR-30a-3p inhibitors** (**single strand)** | Sense: | GCUGCAAACAUCCGACUGAAAG |
| **LINC01436-RT primer (5'-RACE)** |  | CCACCTCCTCATTGAAAAACAGAACACC |
| **LINC01436-R1 (nested PCR )** | Reverse | AGAGGGATGTCTCCAAGATCAGAGCAAA |
| **LINC01436-R2 (nested PCR )** | Reverse | AGAGAAAAGCACGTTAATGCTTTCTGCAA |

**Supplementary Table S4. Univariate and multivariate Cox regression analyses of overall survival in NSCLC patients.**

| **Covariates** | **Univariate analysis** | |  | **Multivariate analysis** | |
| --- | --- | --- | --- | --- | --- |
|  | **HR (95% CI)** | **P-value** |  | **HR (95% CI)** | **P-value** |
| **Age** | 1.019 (1.004-1.034) | **0.014*** |  | 1.028 (1.010-1.047) | **0.003**** |
| **Gender** |  |  |  |  |  |
| Male | Reference |  |  | Reference |  |
| Female | 0.847 (0.649-1.105) | 0.220 |  | 0.872 (0.640-1.188) | 0.384 |
| **Stage** |  |  |  |  |  |
| I | Reference |  |  | Reference |  |
| II | 1.800 (1.298-2.497) | **0.000**** |  | 2.110 (1.452-3.067) | **0.000*** |
| III | 2.664 (1.928-3.682) | **0.000**** |  | 2.611 (1.714-3.976) | **0.000**** |
| IV | 2.197 (1.201-4.020) | **0.011*** |  | 2.399 (1.167-4.930) | **0.017*** |
| **Smoking** |  |  |  |  |  |
| Never smoked | Reference |  |  | Reference |  |
| Smoker | 1.017 (0.776-1.333) | 0.902 |  | 1.143 (0.846-1.544) | 0.386 |
| **Radiation therapy** |  |  |  |  |  |
| No | Reference |  |  | Reference |  |
| Yes | 1.345 (0.894-2.024) | 0.155 |  | 1.306 (0.824-2.071) | 0.255 |
| **Targeted molecular therapy** |  |  |  |  |  |
| No | Reference |  |  | Reference |  |
| Yes | 0.980 (0.695-1.383) | 0.910 |  | 0.671 (0.455-0.991) | **0.045*** |
| **LINC01436 expression level** |  |  |  |  |  |
| Low | Reference |  |  | Reference |  |
| High | 1.321 (1.016-1.718) | **0.038 *** |  | 1.495 (1.102-2.028) | **0.010*** |

HR, hazard ratio; CI, confidence interval. Statistically significant (*P < 0.05 and **P < 0.01).

**Supplementary Table S5. MiRNA expression validation by TCGA datasets**

| **MicroRNA** | **Accession number** | **Expression change in tumor tissues in our study** | **TCGA dataset** | | | | **Consistency with our results** |
| --- | --- | --- | --- | --- | --- | --- | --- |
|  |  |  | **Normal Mean (SD)** | **Tumor Mean (SD)** | **P value** | **Expression change in tumor tissues in TCGA dataset** |  |
| hsa-miR-30a-3p | MIMAT0000088 | down | 14.130 (0.737) | 11.458 (1.223) | 0.000 | down | Yes |
| hsa-miR-486-5p | MIMAT0002177 | down | 9.970 (1.472) | 6.526 (1.510) | 0.000 | down | Yes |
| hsa-miR-486-3p | MIMAT0004762 | down | 1.100 (1.086) | 0.724 (0.502) | 0.003 | down | Yes |
| hsa-miR-139-5p | MIMAT0000250 | down | 6.650 (1.202) | 4.849 (1.087) | 0.000 | down | Yes |
| hsa-miR-27a-5p | MIMAT0004501 | down | 4.872 (1.332) | 3.764 (1.081) | 0.000 | down | Yes |
| hsa-miR-4521 | MIMAT0019058 | down | 0.971 (0.944) | 0.635 (0.395) | 0.002 | down | Yes |
| hsa-miR-338-5p | MIMAT0004701 | down | 4.820 (0.656) | 2.788 (1.240) | 0.000 | down | Yes |
| hsa-miR-133a-3p | MIMAT0000427 | down | 4.504 (1.088) | 2.397 (1.210) | 0.000 | down | Yes |
| hsa-miR-223-3p | MIMAT0000280 | down | 9.082 (1.151) | 8.097 (1.298) | 0.000 | down | Yes |
| hsa-miR-196a-5p | MIMAT0000226 | up | 1.207 (0.797) | 4.613 (2.750) | 0.000 | up | Yes |
| hsa-miR-183-3p | MIMAT0004560 | up | 0.374 (0.237) | 1.443 (0.797) | 0.000 | up | Yes |
| hsa-miR-429 | MIMAT0001536 | up | 5.269 (0.860) | 6.739 (1.223) | 0.000 | up | Yes |
| hsa-miR-96-5p | MIMAT0000095 | up | 2.082 (0.638) | 4.619 (1.110) | 0.000 | up | Yes |
| hsa-miR-4709-3p | MIMAT0019812 | up | 1.805 (0.622) | 1.484 (0.873) | 0.000 | down | No |
| hsa-miR-183-5p | MIMAT0000261 | up | 10.720 (0.562) | 13.341 (1.127) | 0.000 | up | Yes |
| hsa-miR-210-3p | MIMAT0000267 | up | 5.139 (1.824) | 1.008 (1.745) | 0.000 | up | Yes |
| hsa-miR-31-5p | MIMAT0000089 | up | 0.702 (0.572) | 3.087 (2.055) | 0.000 | up | Yes |
| hsa-miR-200a-3p | MIMAT0000682 | up | 6.243 (1.063) | 8.076 (1.272) | 0.000 | up | Yes |
| hsa-miR-205-5p | MIMAT0000266 | up | 5.337 (2.211) | 9.434 (4.273) | 0.000 | up | Yes |
| hsa-miR-3687 | MIMAT0018115 | up | 0.560 (0.651) | 1.150 (0.830) | 0.000 | up | Yes |
| hsa-miR-1273e | MIMAT0018079 | up | Not available |  |  |  | Not available |
| hsa-miR-4538 | MIMAT0019081 | up | Not available |  |  |  | Not available |
| hsa-miR-5096 | MIMAT0020603 | up | Not available |  |  |  | Not available |
| hsa-miR-4417 | MIMAT0018929 | up | Not available |  |  |  | Not available |
| hsa-miR-622 | MIMAT0003291 | up | Not available |  |  |  | Not available |
| hsa-miR-1273d | MIMAT0015090 | up | Not available |  |  |  | Not available |


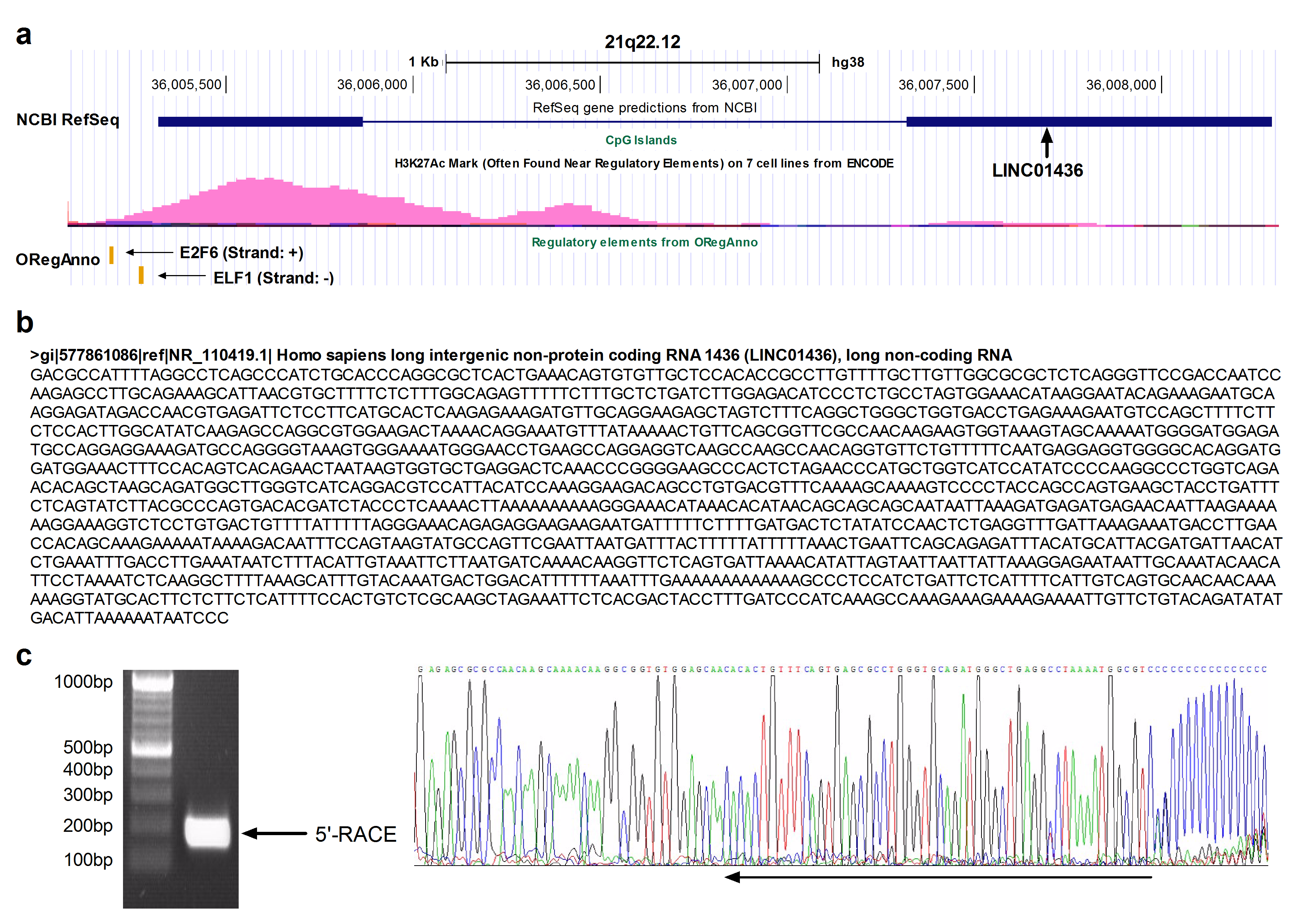


**Supplementary Fig. S1. Genomic location and the full length of LINC01436.** (a) The localization of LINC01436 on the UCSC genome browser. The ORegAnno (Open Regulatory Annotation) track in the UCSC genome browser displays an E2F6 binding site (Strand: +, -292bp ~ -281bp) in the promoter region of LINC01436. (b) The full sequence of LINC01436 (1523 nt). (c) Left: Representative image of PCR products from the 5'-RACE procedure. Right: Sequencing of PCR products revealed the 5’-TSS of LINC01436.


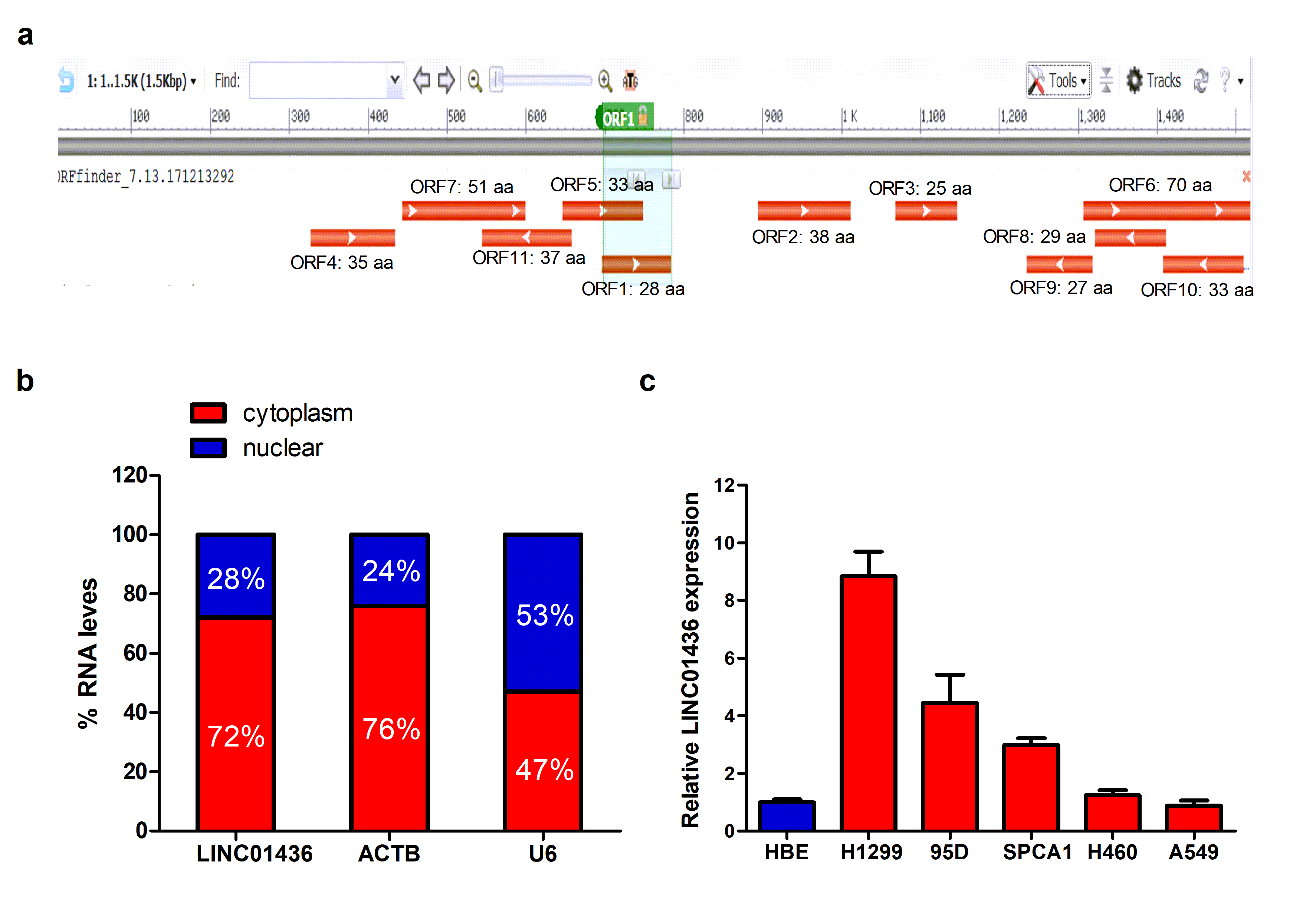
**Supplementary Fig. S2. Potential ORFs and subcellular localization of LINC01436.** (a) ORF prediction of LINC01436 sequence. Eleven potential ORFs that might code peptides of 25 to 70 amino acids are present in the LINC01436 sequence. (b) qRT-PCR detection of the percentage of LINC01436, ACTB and U6 in the nuclear and cytoplasmic fractions of SPCA1 cells. ACTB and U6 serve as a cytoplasmic and nuclear localization control, respectively. (c) qRT-PCR of LINC01436 expression in lung cancer cell lines compared to that in normal human bronchial epithelial cell line (HBE). Error bars represent the SD of three independent experiments.


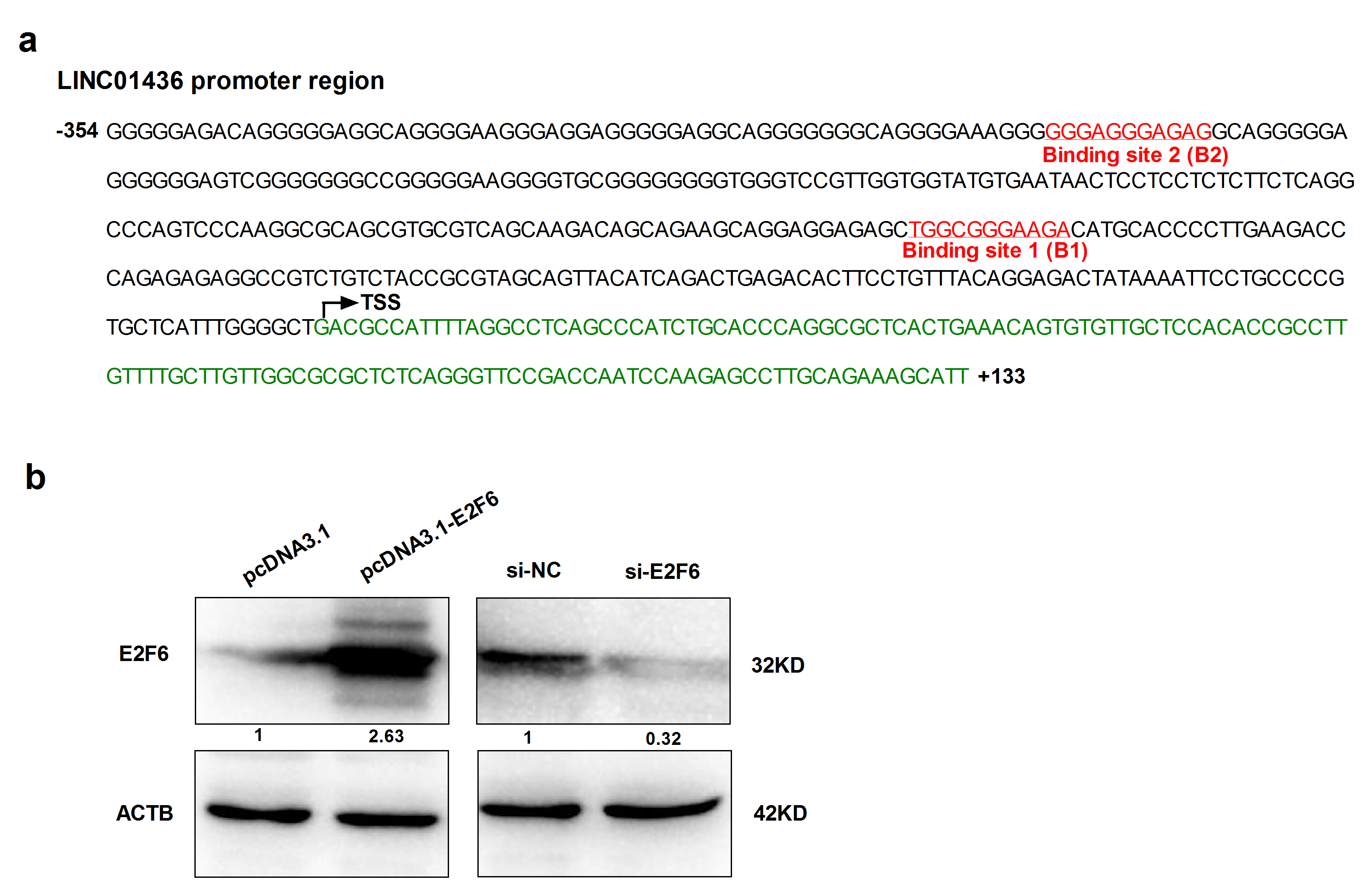


**Supplementary Fig. S3. LINC01436 expression is repressed by E2F6.** (a) Two E2F6 binding sites were predicted in the promoter region of LINC01436. TSS, transcription start site. The promoter sequence (-354bp ~ +133bp) of LINC01436 was synthesized and subcloned into the pGL3-basic vector. (b) The overexpression or knockdown expression of E2F6 was confirmed by western blot in H1299 cells. The numbers in the figure indicate the quantitative analysis results.


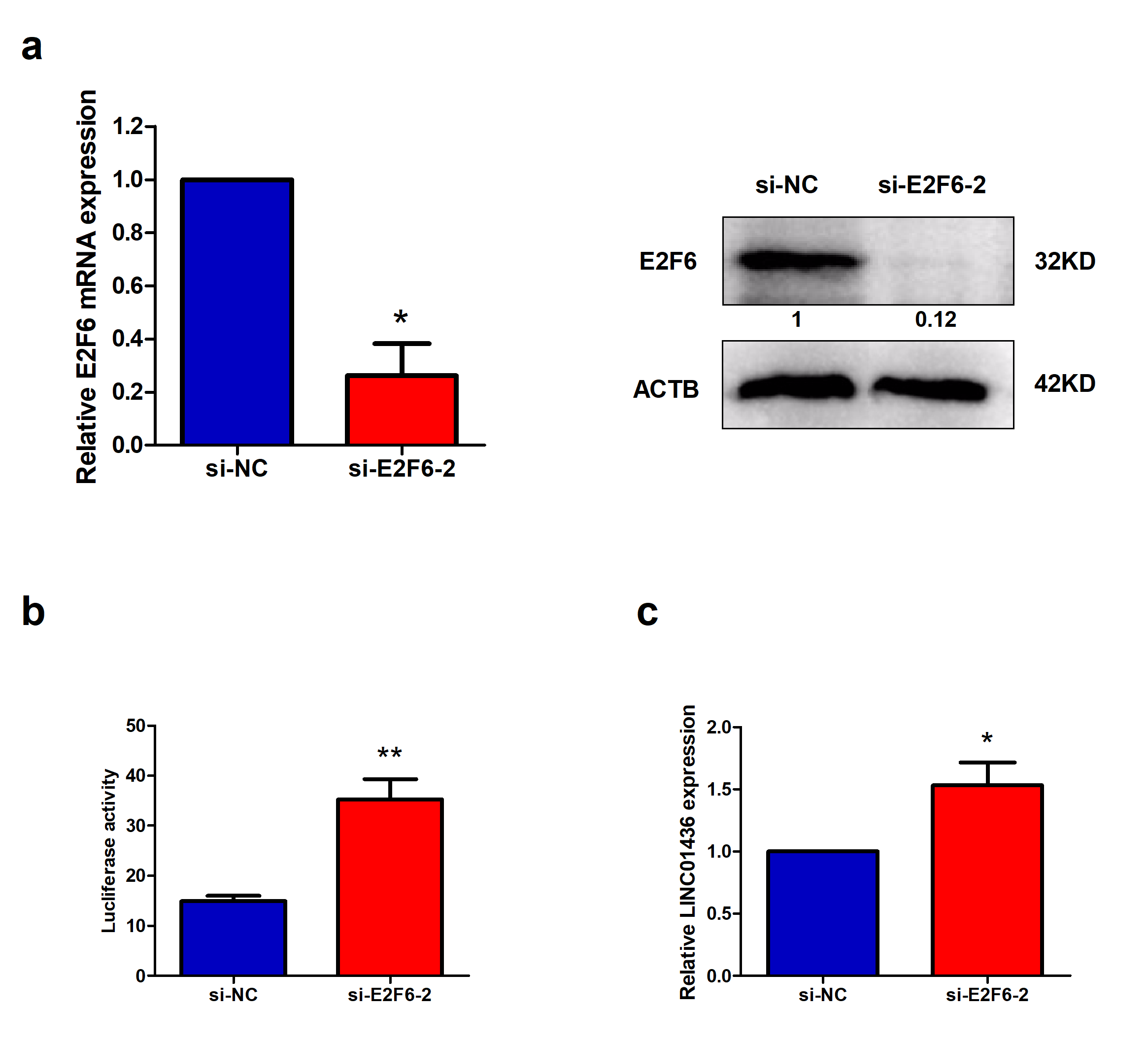


**Supplementary Fig. S4.** **The luciferase activity of LINC01436 promoter and LINC01436 expression in H1299 cells after knockdown of E2F6 by si-E2F6-2.** (a) The knockdown expression of E2F6 by si-E2F6-2 were confirmed by qRT-PCR (left) and western blot (right) in H1299 cells. The numbers in the figure indicate the quantitative analysis results of western blot. (b) Luciferase assays of H1299 cells co-transfected with si-E2F6-2. The LINC01436 promoter sequence (-354bp ~ +133bp) was synthesized and subcloned into the pGL3-basic vector. (c) LINC01436 expression levels in H1299 cells after knockdown of E2F6 by si-E2F6-2. Error bars represent the SD of three independent experiments. * Student’s *t*-test, *P* < 0.05; ** Student’s *t*-test, *P* < 0.01.


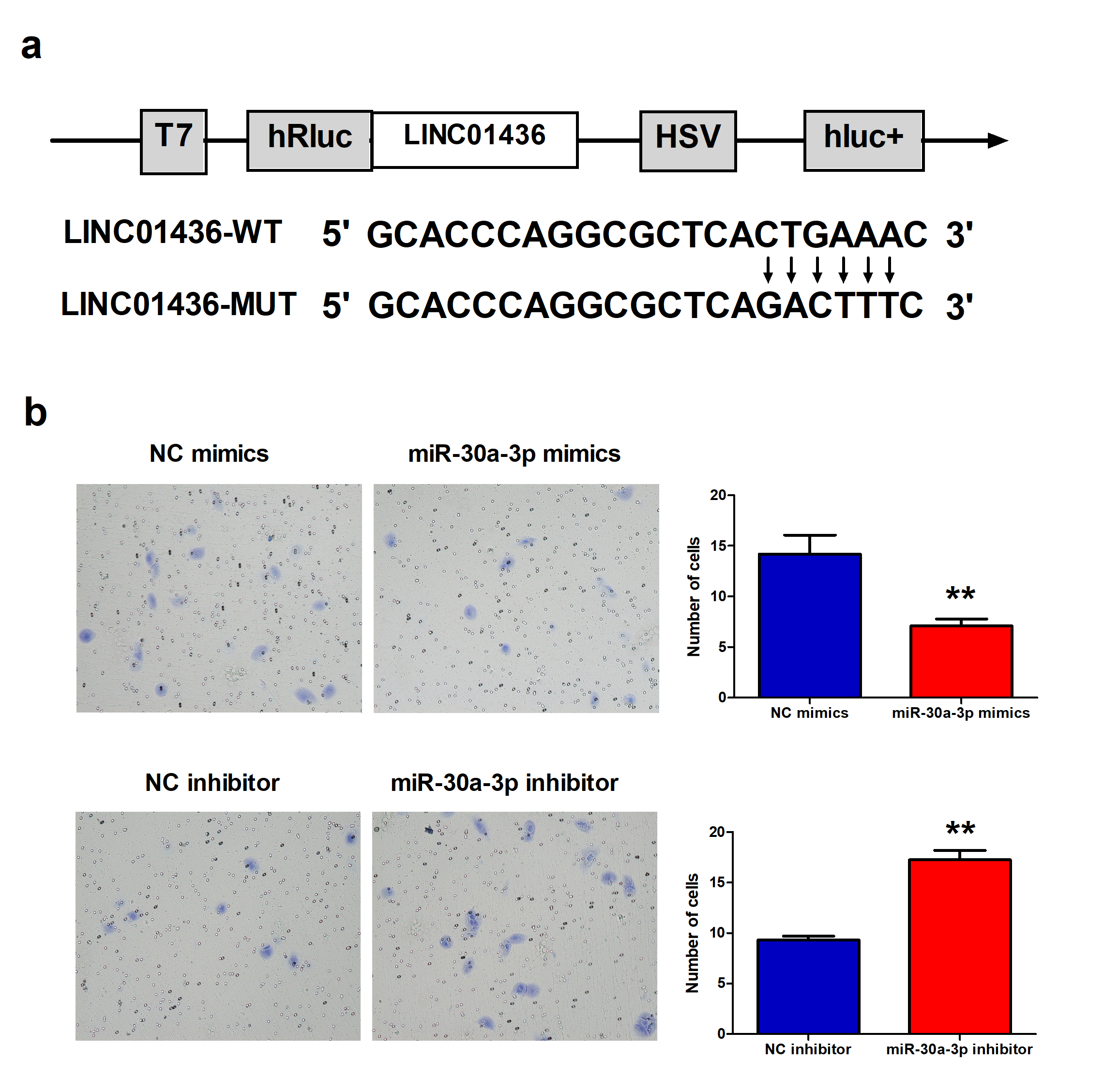
**Supplementary Fig. S5. LINC01436 serves as a sponge for miR-30a-3p and the tumor-suppressive effects of miR-30a-3p in lung cancer cells.** (a) Schematic of LINC01436-WT (wild-type) and LINC01436-MUT (mutant) pmiR-RB-REPORT^TM^ luciferase vectors. (b) Migration of A549 cells transfection with miR-30a-3p inhibitors or miR-30a-3p mimics were determined by transwell assays. Error bars represent the SD of three independent experiments. ** Student’s t-test, *P* < 0.01.


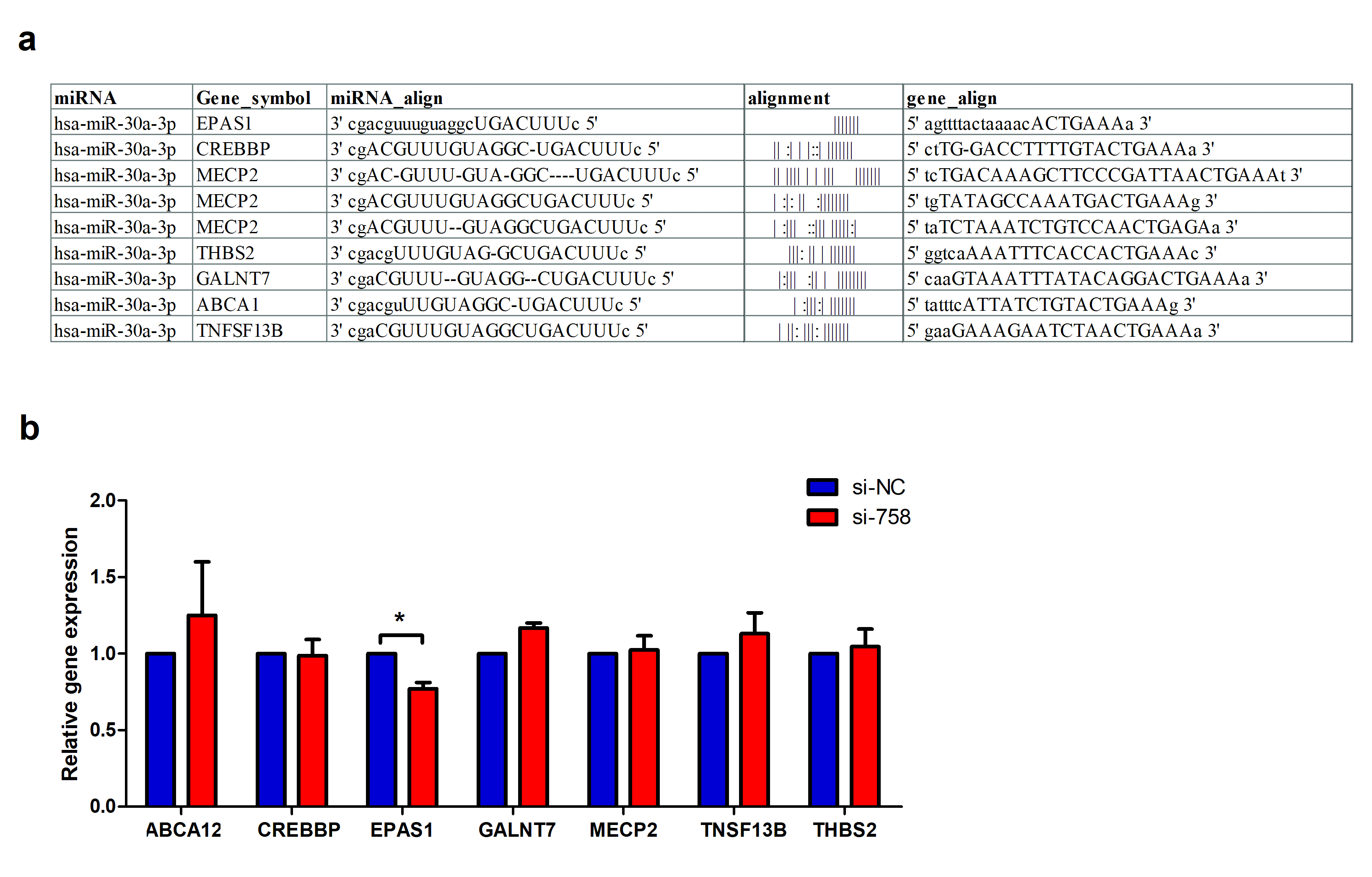


**Supplementary Fig. S6. EPAS1 is a target gene of LINC01436 through miR-30a-3p.** (a) Potential mRNAs were predicted to be targets of miR-30a-3p with miRanda algorithm. (b) Expression levels of potential miR-30a-3p target genes in SPCA1 cells after LINC01436 knockdown. Error bars represent the SD of three independent experiments. * Student’s *t*-test, *P* < 0.05.


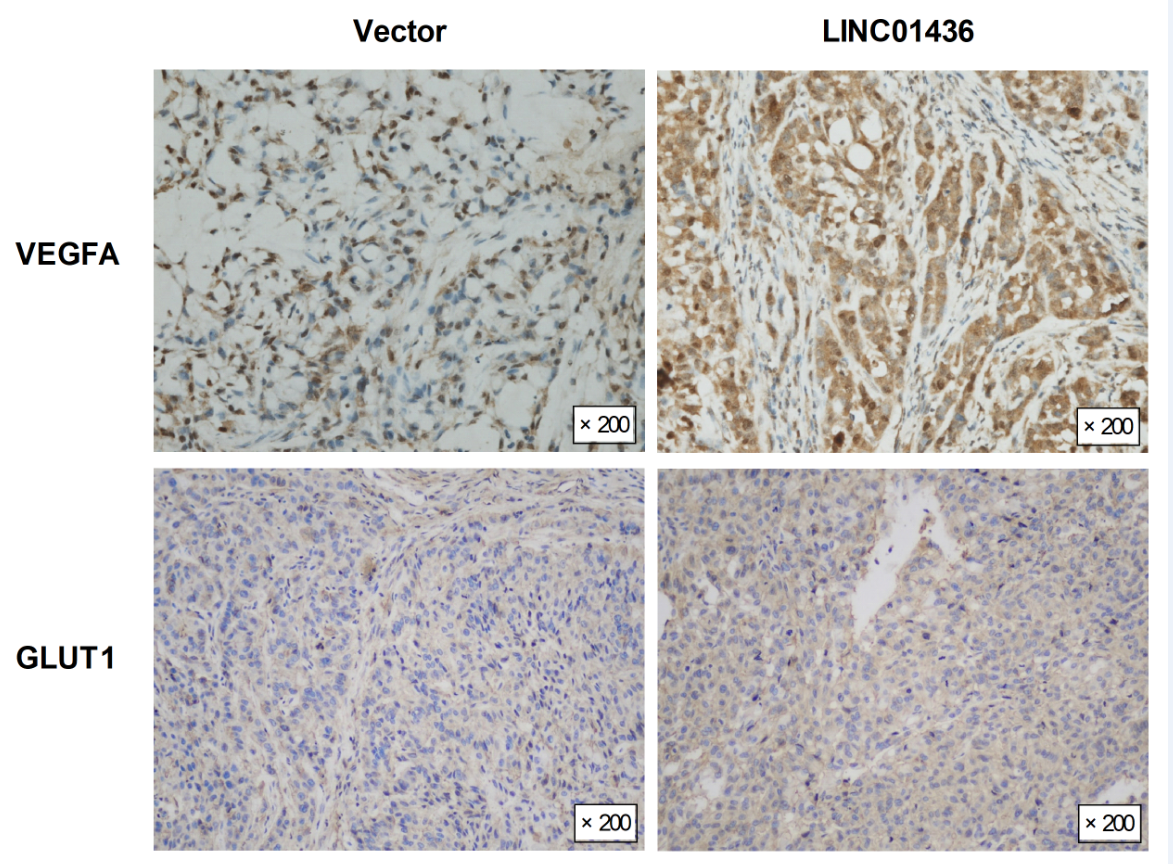


**Supplementary Fig. S7. Immunohistochemical analysis was performed to assess the protein expression levels of EPAS1 target genes (VEGFA and GLUT1).** We have constructed subcutaneous xenograft tumor models using A549 cells stably transfected with LINC01436 or control vector in nude mice in the previous results. We detected the protein expression levels of EPAS1 target genes (VEGFA and GLUT1) through immunohistochemistry in tumor tissues in eight nude mice (four mice in each group). VEGFA and GLUT1 protein expression levels were higher in LINC01436 overexpression A549 cells than those in control vector group. Magnification, ×200.


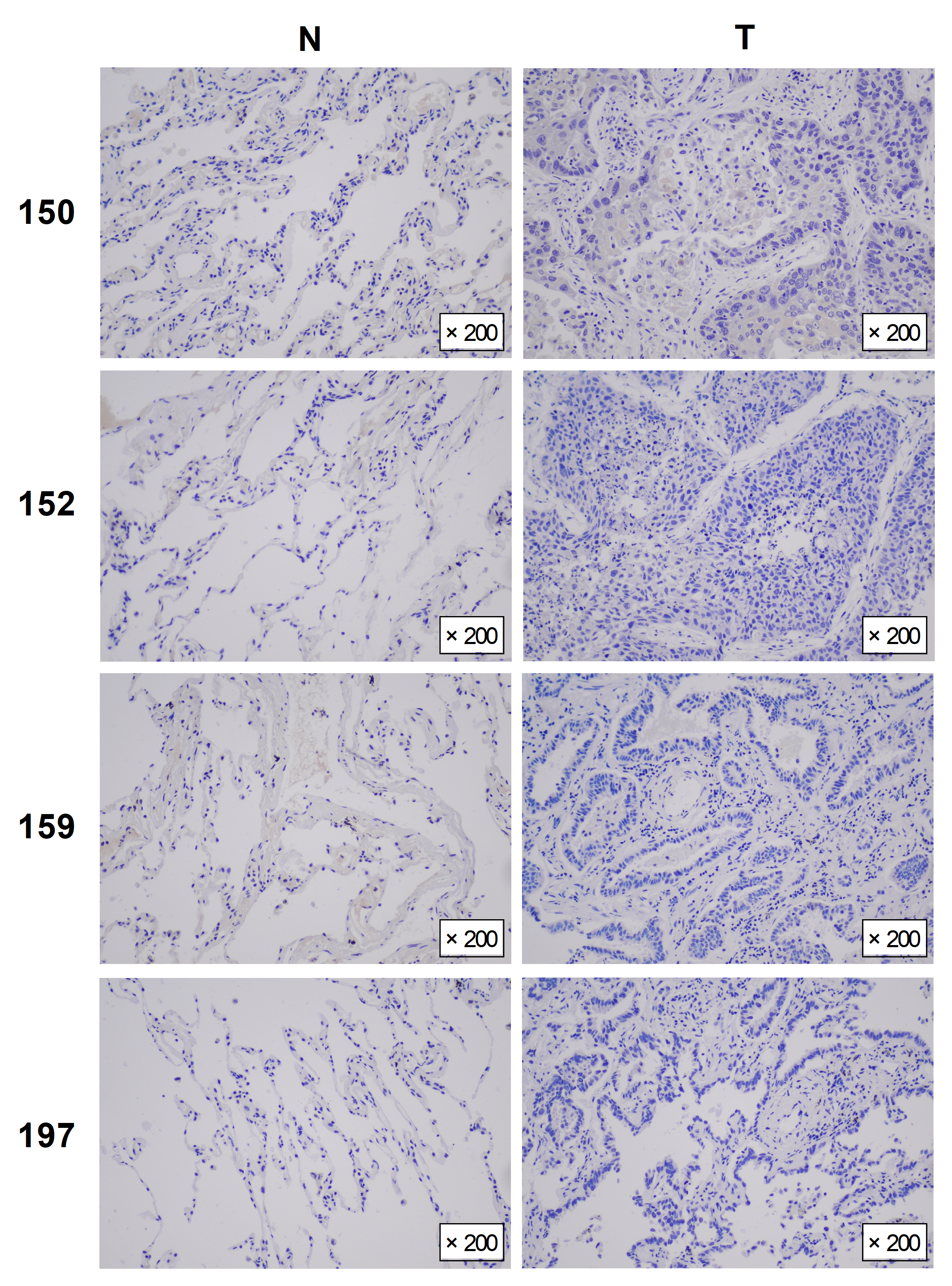


**Supplementary Fig. S8. Immunohistochemical analysis was performed to assess the protein expression levels of EPAS1.** Immunohistochemistry assays were performed to determine the protein expression of EPAS1 in 12 paired NSCLC tissues and adjacent normal lung tissues. However, no difference was found for EPAS1 protein expression between lung tumor tissues and adjacent normal lung tissues. 4 paired representative immunohistochemical staining were present. T, tumor tissue; N, normal tissue. Magnification, ×200.
